# Supplementary material for: The lived experience of long COVID: A thematic analysis of an in-depth interview study
Source: PLOS Ment Health. 2026 Feb 6;3(2):e0000500. doi: 10.1371/journal.pmen.0000500 (PMC12880701; doi:10.1371/journal.pmen.0000500)
Supplement: S1 Table — (DOCX) [file pmen.0000500.s001.docx]

**S1 Table. Acute Infection Codes**

| **Code:** | **Code Endorsement Range:** | **Code Description:** | **Example Quotes:** |
| --- | --- | --- | --- |
| **Acute Sars-CoV-2 Infection** |  |  |  |
| **Number of Infections** |  |  |  |
| 4+ | 3 (8.8%) | Four or more acute COVID-19 infections (suspected or confirmed). | “So I would say I've had four rounds of COVID.” |
| 3 | 3 (8.8%) | Three acute COVID-19 infections (suspected or confirmed). | “I've had two for sure, where I tested positive.  And I believe I also had one more before the tests were readily available." |
| 2 | 8 (23.4%) - 9 (26.5%0 | Two acute COVID-19 infections (suspected or confirmed). | “I've had two. The first was in December of 2020. The second was in February of this last year, 2023.” |
| 1 | 18 (52.9%) | One acute COVID-19 infections (suspected or confirmed). | “I've only tested positive on a test once, and it was basically whenever like the month before my long COVID symptoms started.” |
| **Trajectory to Chronic Symptoms** |  |  |  |
| Never Improved | 14 (41.2%) - 15 (44.1%) | Symptoms never improved from acute COVID-19 infection and transitioned into LC symptoms. | “So I took it really easy for those three weeks because I was still pretty sick. About week four, when nothing was getting better, I kind of started thinking crap, this might be that long COVID that people are talking about.” |
| Worsened over time | 1 (2.9%) - 4 (11.8%) | Symptoms worsened over time from acute COVID-19 infection to LC symptoms. | “It just slowly got worse and worse...” |
| Improved from initial infection before worsening | 15 (44.1%) - 17 (50.0%) | Symptoms improved from acute COVID-19 infection and subsequentially worsened into LC symptoms. | “Actually, I got better… after I was infected for about a month and then I started getting long-term effects.” |
| Each infection brought new LC symptoms | 5 (14.7%) - 7 (20.6%) | New symptoms developed from each acute COVID-19 infection and developed into LC symptoms. | “It seems like every time I pick up a new symptom.” |
| First infection caused long COVID | 22 (64.7%) - 23 (67.6%) | LC symptoms developed from first acute COVID-19 infection. | “My first infection, I did not recover from. I would consider it long COVID.” |
| Later infections caused long-term symptoms | 3 (8.8%) - 5 (14.7%) | LC symptoms developed from acute COVID-19 infections after the initial infection period. | “And this last one was what has really affected my life.” |
| **Treatment Sought** |  |  |  |
| Intensive care unit (ICU) | 1 (2.9%) | Treatment was received at an ICU for an acute COVID-19 infection. | “I was in our local community hospital, either in ICU or in whatever the next layer below that is.” |
| ER | 6 (17.6%) - 7 (20.6%) | Treatment was received at an ER for an acute COVID-19 infection. | “I did actually for that one, go to the ER at the end of July, I think, because I started having chest pain, really bad chest pain and cramping in my legs.” |
| Urgent/Express care | 3 (8.8%) - 4 (11.8%) | Treatment was received at an Urgent or Express Care for an acute COVID-19 infection. | “I went right to urgent care and I was like, I think, I think I have it.” |
| Primary care | 7 (20.6%) | Treatment was received from a primary care provider for an acute COVID-19 infection. | “September of that same year, I went to my GP and she said, okay, you know, we'll send you for testing.” |
| Specialist | 2 (5.9%) | Treatment was received from a specialist for an acute COVID-19 infection. | “And it was like she was sending me to the ear, nose and throat or the rheumatologist or all these places where I had… symptoms.” |
| Unclear | 9 (26.5%) - 10 (29.4%) | Treatment was received for an acute COVID-19 infection, although the source/type of provider is unclear. | “And I had seen a couple of doctors by then and everybody was kind of just telling me like, this seems like it's in your head.” |
| Medications For Acute COVID | 13 (38.2%) - 14 (41.2%) | Medication was taken for an acute COVID-19 infection. | “I had the monoclonal antibody infusion at the hospital. And I also attempted to do the trial of Paxlovid.” |
| **Initial Infection Severity** |  |  |  |
| Severe | 9 (26.5%) - 10 (29.4%) | Acute COVID-19 infection(s) was(were) described as a severe infection/illness. | “The first infection I would describe as severe.” |
| Moderate | 6 (17.6%) - 8 (23.5%) | Acute COVID-19 infection(s) was(were) described as a moderate infection/illness. | “I would say, I'd say like on a scale of one to ten, I'd probably give it like a six or a seven with ten being like the most severe thing I've ever experienced.” |
| Mild | 10 (29.4%) - 12 (35.3%) | Acute COVID-19 infection(s) was(were) described as a mild infection/illness. | “My second infection was super mild.” |
| **Distinct from Other Illness** |  |  |  |
| No | 8 (23.5%) - 9 (26.5%) | Acute COVID-19 infection was not distinct/distinguishable from other illnesses (cold/flu/etc.) | “I felt basically the whole time like I was getting over a bad case of the flu.” |
| Yes | 18 (52.9%) - 20 (58.8%) | Acute COVID-19 infection was distinct/distinguishable from other illnesses (cold/flu/etc.) due to worse/more severe/different symptoms. | “It wasn't like anything I have ever had before. I mean, I've had colds and I've not felt well (but) this took me down and out.” |
| Negative Reaction to Vaccine | 5 (14.7%) | A negative reaction to a COVID-19 vaccine was experienced. | “I couldn't get the second vaccine… because I had a really bad reaction to the first one.” |
| **Initial Infection Length** |  |  |  |
| >2 weeks | 10 (29.4%) - 11 (32.4%) | Acute COVID-19 infection lasted for over two weeks. | “So my first infection, I was down for about three weeks. So I was very pretty sick for about three weeks.” |
| 1-2 weeks | 12 (35.3%) - 15 (44.1%) | Acute COVID-19 infection lasted for 1-2 weeks. | “And I would say that was probably about a week, week and a half of that before it let up any, but it did.” |
| <1 week | 3 (8.8%) - 6 (17.6%) | Acute COVID-19 infection lasted for less than 1 week. | “My second infection, I would say three days, maybe three days.” |
| **Initial Infection Symptoms** |  |  |  |
| Dehydration | 1 (2.9%) - 3 (8.8%) | Reported experiencing dehydration during an acute COVID-19 infection | “I went into the emergency room because I dehydrated.” |
| Sore throat | 6 (17.6%) - 7 (20.6%) | Reported experiencing sore throat during an acute COVID-19 infection | “It was just a sore throat for a couple of days.” |
| Chest congestion | 3 (8.8%) - 5 (14.7%) | Reported experiencing chest congestions during an acute COVID-19 infection | “So it was just cold symptoms, stuffed up nose, scratchy throat, some congestion in the chest.” |
| Blood pressure issues | 1 (2.9%) | Reported experiencing blood pressure issues during an acute COVID-19 infection | “… it seemed like my blood pressure was really high.” |
| Vomiting | 2 (5.9%) | Reported experiencing vomiting during an acute COVID-19 infection | “That was … three days of fever, diarrhea, and vomiting...” |
| Nausea | 4 (11.8%) | Reported experiencing nausea during an acute COVID-19 infection | “It actually started with nausea.” |
| Hearing changes | 1 (2.9%) | Reported experiencing hearing changes during an acute COVID-19 infection | “I lost my peripheral vision and hearing in my left ear.” |
| Vision changes | 1 (2.9%) | Reported experiencing vision changes during an acute COVID-19 infection | “I lost my peripheral vision and hearing in my left ear.” |
| Losing smell or taste | 11 (32.4%) | Reported experiencing loss of smell and/or taste during an acute COVID-19 infection | “But I lost taste and smell” |
| Joint pain | 3 (8.8%) - 5 (14.7%) | Reported experiencing joint pain during an acute COVID-19 infection | “The muscle pain was all over the body, but in the joints.” |
| Balance difficulties | 0 (0.0%) - 1 (2.9%) | Reported experiencing balance difficulties during an acute COVID-19 infection | “I felt a little like off balance...” |
| Body aches | 9 (26.5%) - 13 (38.2%) | Reported experiencing body aches during an acute COVID-19 infection | “It really started with the body aches and the headache and then it went into the tiredness.” |
| Sinus/head congestion/pain | 18 (52.9%) - 21 (61.8%) | Reported experiencing sinus/head congestion and/or pain during an acute COVID-19 infection | “Congested, but like, not running. So like, nothing would come out. It was all stuck in my face.” |
| Fever | 10 (29.4%) - 11 (32.4%) | Reported experiencing fever during an acute COVID-19 infection | “I had a high fever the day before I had an idea of what it was.” |
| Headaches/migraine | 14 (41.2%) - 16 (47.1%) | Reported experiencing headaches and/or migraines during an acute COVID-19 infection | “And also I had severe headaches.” |
| Coughing | 17 (50.0%) - 18 (52.9%) | Reported experiencing coughing during an acute COVID-19 infection | “I was coughing constantly or sleeping for about a week and a half before I began to improve.” |
| Difficulty breathing | 9 (26.5%) - 10 (29.4%) | Reported experiencing difficulty breathing during an acute COVID-19 infection | “And I know that at times my oxygen levels fell to the eighties and, um, I was just, I was struggling to breathe.” |
| Muscle soreness | 4 (11.8%) - 5 (14.7%) | Reported experiencing muscle soreness during an acute COVID-19 infection | “At first it was just kind of muscle soreness.” |
| Cognitive issues | 5 (14.7%) - 7 (20.6%) | Reported experiencing cognitive issues during an acute COVID-19 infection | “And I lived in a three story house at the time and I was having so much trouble with dementia that I couldn't remember where anything was.” |
| Fatigue | 20 (58.8%) | Reported experiencing fatigue during an acute COVID-19 infection | “Just really, really extreme fatigue.” |
| Night sweats/sweating | 4 (11.8%) | Reported experiencing night sweats/sweating during an acute COVID-19 infection | “I literally would soak the sheets on the bed just from sweating.” |
| Chills | 0 (0.0%) - 2 (5.9%) | Reported experiencing chills during an acute COVID-19 infection | “So the first symptom, I was like laying in bed about to go to sleep, and I started shaking uncontrollably, and I felt really cold.” |
| Diarrhea | 1 (2.9%) - 2 (5.9%) | Reported experiencing diarrhea during an acute COVID-19 infection | “I didn't have a solid bowel movement for six weeks.” |
| Cold/couldn't warm up | 2 (5.9%) | Reported experiencing coldness/inability to warm up during an acute COVID-19 infection | “It was just like, all of a sudden, I was just cold, and I could not get warm.” |
| Kidney issues | 0 (0.0%) - 2 (5.9%) | Reported experiencing kidney issues during an acute COVID-19 infection | “Yeah, I had kidney failure.” |
| Blisters on tongue | 0 (0.0%) - 1 (2.9%) | Reported experiencing blisters on the tongue during an acute COVID-19 infection | “I've got these blisters on my tongue.” |
| Runny nose | 1 (2.9%) - 6 (17.6%) | Reported experiencing running nose during an acute COVID-19 infection | “Oh, it was like a runny nose and, you know, a little bit of a scratchy throat.” |
| Low oxygen level | 1 (2.9%) - 2 (5.9%) | Reported experiencing low oxygen levels during an acute COVID-19 infection | “And on the fifth or sixth day, I noticed my pulse ox dropping pretty quickly.” |
| Dizziness/lightheaded/vertigo | 1 (2.9%) - 2 (5.9%) | Reported experiencing dizziness/lightheadedness/vertigo during an acute COVID-19 infection | “Well, it was like about three days after I got diagnosed with the COVID that I started with the vertigo.” |
| Edema/swelling | 1 (2.9%) - 2 (5.9%) | Reported experiencing edema and/or swelling during an acute COVID-19 infection | “I was having edema and I couldn't wear shoes.” |
| Abnormal clotting | 1 (2.9%) | Reported experiencing abnormal clotting during an acute COVID-19 infection | “If I cut myself, the blood seemed to be very thick and it would just clot immediately.” |
| Rash | 1 (2.9%) - 2 (5.9%) | Reported experiencing rash during an acute COVID-19 infection | “And then there were the rashes, strange rashes. There was one across my upper torso and then there were some on my ankles. And then the blood vessels started popping.” |
| Heart rate change | 2 (5.9%) - 3 (8.8%) | Reported experiencing heart rate change during an acute COVID-19 infection | “I had just sort of heart palpitations, sweats.” |
| Malaise | 9 (26.5%) - 10 (29.4%) | Reported experiencing malaise during an acute COVID-19 infection | “Just, you know, that sort of heavy, I'm sick sort of feeling like you just can't, you can do a few things, but you just can't do that much.” |
