# Supplementary material for: The lived experience of long COVID: A thematic analysis of an in-depth interview study
Source: PLOS Ment Health. 2026 Feb 6;3(2):e0000500. doi: 10.1371/journal.pmen.0000500 (PMC12880701; doi:10.1371/journal.pmen.0000500)
Supplement: S2 Table — (DOCX) [file pmen.0000500.s002.docx]

**S2 Table. Comorbid Conditions Codes**

| **Code:** | **Code Endorsement Range:** | **Code Description:** | **Example Quotes:** |
| --- | --- | --- | --- |
| **Comorbid Conditions** |  |  |  |
| Cancer | 0 (0.0%) | Previous/Comorbid/Subsequent diagnosis of cancer | N/A |
| Restless leg syndrome | 1 (2.9%) | Previous/Comorbid/Subsequent diagnosis of restless leg syndrome | “Also, growing up, I had restless leg syndrome.” |
| Hernia | 0 (0.0%) - 1 (2.9%) | Previous/Comorbid/Subsequent diagnosis of a hernia | “I have had some issues… it's due to a couple of hernia repairs that I've had issues with.” |
| Allergies | 2 (5.9%) - 3 (8.8%) | Previous/Comorbid/Subsequent diagnosis of allergies | “I had about maybe two sinus infections a year just because of allergies.” |
| Migraine disorder | 1 (2.9%) - 3 (8.8%) | Previous/Comorbid/Subsequent diagnosis of a migraine disorder | “Then I have these cool things that are called ocular migraines now that I never had prior.” |
| Chronic dizziness | 1 (2.9%) | Previous/Comorbid/Subsequent diagnosis of chronic dizziness | “Dizziness for the last five years.” |
| Scoliosis | 1 (2.9%) | Previous/Comorbid/Subsequent diagnosis of scoliosis | “My doctor seems to be more interested in my sleep apnea and my back pain and my scoliosis and stuff.” |
| BPPV | 1 (2.9%) | Previous/Comorbid/Subsequent diagnosis of BPPV | “I had BBPV and then it's been complicated with some vision problems and hearing problems and inner ear situations.” |
| Seasonal affective disorder | 0 (0.0%) - 1 (2.9%) | Previous/Comorbid/Subsequent diagnosis of seasonal affective disorder | “I think I have a little bit of seasonal affective disorder.” |
| Bowel obstruction | 1 (2.9%) | Previous/Comorbid/Subsequent diagnosis of bowel obstruction | “I have had issues with ongoing bowel obstructions…” |
| Meniere’s disease | 1 (2.9%) | Previous/Comorbid/Subsequent diagnosis of Meniere’s disease | “And one of the other things I have is what they call Meniere’s disease...” |
| Unspecified/other chronic pain | 0 (0.0%) - 1 (2.9%) | Previous/Comorbid/Subsequent diagnosis of unspecified/other chronic pain | “My doctor seems to be more interested in my sleep apnea and my back pain and my scoliosis and stuff.” |
| Sleep apnea | 2 (5.9%) | Previous/Comorbid/Subsequent diagnosis of sleep apnea | “My sleep apnea, I've been with my CPAP machine probably 20, 25 years.” |
| Barrett's esophagus | 1 (2.9%) | Previous/Comorbid/Subsequent diagnosis of Barrett’s esophagus | “And have Barrett’s esophagus and then sleep apnea and cardiovascular disease.” |
| Ulcerative colitis | 1 (2.9%) | Previous/Comorbid/Subsequent diagnosis of ulcerative colitis | “I have ulcerative colitis.” |
| Agoraphobia | 1 (2.9%) | Previous/Comorbid/Subsequent diagnosis of agoraphobia | “I was diagnosed with PTSD and I have agoraphobia.” |
| Bipolar | 0 (0.0%) - 1 (2.9%) | Previous/Comorbid/Subsequent diagnosis of bipolar disorder | “… bipolar that I was diagnosed with when I was like, early 20s” |
| Dyslexia | 1 (2.9%) | Previous/Comorbid/Subsequent diagnosis of dyslexia | “I've had dyslexia and a couple of different things...” |
| Neurodiversity | 1 (2.9%) | Previous/Comorbid/Subsequent diagnosis of neurodiversity | “And then there's also just the research and all the stuff I've done to figure out neurodiversity and understand the differences and how I function different than others.” |
| Scleritis | 0 (0.0%) - 1 (2.9%) | Previous/Comorbid/Subsequent diagnosis of scleritis | “It was like, well, you (might have) scleritis.” |
| Large cell arteritis | 0 (0.0%) - 1 (2.9%) | Previous/Comorbid/Subsequent diagnosis of large cell arteritis | “So it's kind of like a large cell arteritis.” |
| Polymyalgia rheumatica (PMR) | 1 (2.9%) | Previous/Comorbid/Subsequent diagnosis of PMR | “I may have been predisposed genetically speaking for this thing called PMR, which is polymyalgia rheumatica.” |
| MGUS (monoclonal gammopathy of undetermined significance) | 1 (2.9%) | Previous/Comorbid/Subsequent diagnosis of MGUS | “They agreed that it was MGUS.” |
| Orthostatic syncope | 1 (2.9%) | Previous/Comorbid/Subsequent diagnosis of orthostatic syncope | “… orthostatic syncope, I think is what my cardiologist called it.” |
| Interstitial cystitis | 0 (0.0%) - 1 (2.9%) | Previous/Comorbid/Subsequent diagnosis of interstitial cystitis | “I had a condition of the bladder called interstitial cystitis.” |
| Asthma | 4 (11.8%) - 5 (14.7%) | Previous/Comorbid/Subsequent diagnosis of asthma | “I've had asthma since I was a kid.” |
| Thyroid disorder | 2 (5.9%) - 3 (8.8%) | Previous/Comorbid/Subsequent diagnosis of thyroid disorder | “I found out that I have low thyroid, but my mom has it, so that might be genetic.” |
| Abnormal intercranial pressure | 1 (2.9%) | Previous/Comorbid/Subsequent diagnosis of abnormal intercranial pressure | “And then we found out it was the thiamin deficiency and the intracranial pressure causing some of the problems.” |
| Thiamine deficiency | 1 (2.9%) | Previous/Comorbid/Subsequent diagnosis of thiamine deficiency | “And then we found out it was the thiamin deficiency and the intracranial pressure causing some of the problems.” |
| Parkinson's characteristics/disease | 1 (2.9%) | Previous/Comorbid/Subsequent diagnosis of Parkinson’s’ characteristics and/or disease | “I got some Parkinson's characteristics now.” |
| ME/CFS (myalgic encephalomyelitis/chronic fatigue syndrome) | 4 (11.8%) | Previous/Comorbid/Subsequent diagnosis of ME and/or CFS | “And I did get a diagnosis of chronic fatigue, ME/CFS, as well as fibromyalgia and long COVID and all the other things I've mentioned so far.” |
| Ehlers-Danlos (EDS)/connective tissue disorder | 4 (11.8%) | Previous/Comorbid/Subsequent diagnosis of Ehlers-Danlos and/or a connective tissue disorder | “I do now know that we have EDS.” |
| Liver problems | 1 (2.9%) | Previous/Comorbid/Subsequent diagnosis of liver problems | “My liver enzymes spiked. I had to see, you know, a liver doctor.” |
| High cholesterol | 3 (8.8%) - 4 (11.8%) | Previous/Comorbid/Subsequent diagnosis of high cholesterol | “I was diagnosed with overly high cholesterol.” |
| Diabetes | 1 (2.9%) | Previous/Comorbid/Subsequent diagnosis of diabetes | “I was diagnosed with diabetes.” |
| Small fiber neuropathy | 1 (2.9%) | Previous/Comorbid/Subsequent diagnosis of small fiber neuropathy | “I have small fiber neuropathy.” |
| Other gastrointestinal condition | 7 (20.6%) - 9 (26.5%) | Previous/Comorbid/Subsequent diagnosis of a GI condition | “I have GI symptoms that aren't super obvious. Like there's no pain, but it's also like stuff is not normal in that vicinity.” |
| Chronic Lyme | 1 (2.9%) - 2 (5.9%) | Previous/Comorbid/Subsequent diagnosis of chronic Lyme | “And we did find out through PCR testing in December that I had active Lyme. So she believes that I have chronic Lyme.” |
| Adrenal insufficiency | 1 (2.9%) | Previous/Comorbid/Subsequent diagnosis of adrenal insufficiency | “And so I had some adrenal insufficiency...” |
| Obesity/overweight | 3 (8.8%) - 6 (17.6%) | Previous/Comorbid/Subsequent diagnosis of being overweight or obese | “I'm overweight. I've always been overweight.” |
| Stenosis of spine | 1 (2.9%) | Previous/Comorbid/Subsequent diagnosis of stenosis of the spine | “I have lumbar and cervical stenosis.” |
| **Arthritis** |  |  |  |
| Unclear/other type | 2 (5.9%) - 6 (17.6%) | Previous/Comorbid/Subsequent diagnosis of arthritis (unclear type) | “They think it's reactive arthritis, so they put me on some medications for that.” |
| Rheumatoid arthritis | 2 (5.9%) | Previous/Comorbid/Subsequent diagnosis of Rheumatoid arthritis | “I didn't start to get the rheumatoid flares until about a year into it.” |
| Osteoarthritis | 0 (0.0%) - 5 (14.7%) | Previous/Comorbid/Subsequent diagnosis of osteoarthritis | “And I do have arthritis. You know, I've had my knees replaced, but it doesn't seem like that kind of joint pain that's related to arthritis.” |
| Herpes | 1 (2.9%) | Previous/Comorbid/Subsequent diagnosis of Herpes | “And then also the herpes one is latent in me as well.” |
| Epstein-Barr/Chronic Mono | 1 (2.9%) | Previous/Comorbid/Subsequent diagnosis of Epstein Barr and/or Chronic Mono | “But I did, once I kind of cleared the medicine out of my system, and we found out it was actually… reactivated Epstein-Barr.” |
| Autoimmune disorder | 2 (5.9%) - 3 (8.8%) | Previous/Comorbid/Subsequent diagnosis of an autoimmune disorder | “But that's all because whatever is going on inside me with the autoimmune stuff is, I guess, causing that.” |
| ADD/ADHD | 5 (14.7%) | Previous/Comorbid/Subsequent diagnosis of ADHD/ADD | “I (have a) diagnosis (of) ADHD and anxiety.” |
| Anxiety/Panic Attack | 12 (35.3%) - 14 (41.2%) | Previous/Comorbid/Subsequent diagnosis of anxiety and/or anxiety attacks | “I started getting anxiety and panic attacks in the summer and (they) made it very hard to do anything.” |
| Depression | 9 (26.5%) - 10 (29.4%) | Previous/Comorbid/Subsequent diagnosis of depression | “Depression is here 24-7. It doesn't matter how I'm feeling. I feel depressed.” |
| Hypertension/HTN | 5 (14.7%) - 6 (17.6%) | Previous/Comorbid/Subsequent diagnosis of hypertension | “And hypertension as well.” |
| Postural orthostatic tachycardia syndrome (POTS)/Dysautonomia/Orthostatic intolerance | 4 (11.8%) - 7 (20.6%) | Previous/Comorbid/Subsequent diagnosis of POTS/Dysautonomia/Orthostatic intolerance | “I still have POTS, but I've learned how to manage it with meditation.” |
| Fibromyalgia | 1 (2.9%) | Previous/Comorbid/Subsequent diagnosis of fibromyalgia | “And I did get a diagnosis of chronic fatigue, ME/CFS, as well as fibromyalgia and long COVID and all the other things I've mentioned so far.” |
| Heart issues/disease | 2 (5.9%) - 7 (20.6%) | Previous/Comorbid/Subsequent diagnosis of heart issues and/or disease | “I did have arrhythmia so my heart would just start beating really fast up and down.” |
| Kidney failure | 0 (0.0%) - 1 (2.9%) | Previous/Comorbid/Subsequent diagnosis of kidney failure | “Yeah, I had kidney failure.” |
| Lung damage/disease | 3 (8.8%) | Previous/Comorbid/Subsequent diagnosis of lung damage and/or disease | “I later found out it's connective tissue around my lungs that doesn't expand.” |
| Atrial fibrillation | 1 (2.9%) - 2 (5.9%) | Previous/Comorbid/Subsequent diagnosis of atrial fibrillation | “The doctor says more like AFib.” |
| Brain tumor | 0 (0.0%) - 1 (2.9%) | Previous/Comorbid/Subsequent diagnosis of a brain tumor | “All of this is complicated, though, because in the midst of doing all of the testing to figure out what was going on with me, they discovered that the headaches that I'd been having were caused by a small tumor in the lining of my brain that's obviously been there a long time, but it's only recently diagnosed, and I don't know yet what we're going to do about that.” |
| Mast cell activation | 3 (8.8%) | Previous/Comorbid/Subsequent diagnosis of mast cell activation | “I've been diagnosed with mast cell activation, which it fits with some of the symptoms I had before I got COVID but has been exacerbated to the point where it's undeniable.” |
| Vascular issues | 1 (2.9%) | Previous/Comorbid/Subsequent diagnosis of vascular issues | “They did a CT, found the aneurysm...” |
| Lymphatic issues | 0 (0.0%) -1 (2.9%) | Previous/Comorbid/Subsequent diagnosis of lymphatic issues | “(I have a) very poor lymphatic drainage system and so you're sort of never disposing of waste properly and then sort of it's like this one thing that breaks the camel's back.” |
| Bronchitis | 0 (0.0%) - 1 (2.9%) | Previous/Comorbid/Subsequent diagnosis of bronchitis | “Well, I had had bronchitis for a while and was trying to get over it and so I was feeling poorly, but just realizing that it's my normal time for getting bronchitis and went in to get some treatment for the bronchitis and the doctor said, well, let's go ahead and check and see if you have COVID and it came back positive.” |
| Osteoporosis | 1 (2.9%) | Previous/Comorbid/Subsequent diagnosis of osteoporosis | “I just suffer from osteoporosis.” |
