# Supplementary material for: The lived experience of long COVID: A thematic analysis of an in-depth interview study
Source: PLOS Ment Health. 2026 Feb 6;3(2):e0000500. doi: 10.1371/journal.pmen.0000500 (PMC12880701; doi:10.1371/journal.pmen.0000500)
Supplement: S3 Table — (DOCX) [file pmen.0000500.s003.docx]

**S3 Table. COVID Susceptibility Codes**

| **Code:** | **Code Endorsement Range:** | **Code Description:** | **Example Quotes:** |
| --- | --- | --- | --- |
| **Personal Susceptibility Perception** |  |  |  |
| **Long COVID** |  |  |  |
| **No** |  |  |  |
| Careful | 13 (38.2%) - 14 (41.2%) | Did not feel a personal susceptibility to LC due to living carefully/practicing safety precautions/following LC health guidelines prior to developing LC | “I mean we took the precautions and right when vaccinations were available to the mainstream public that's right when I got it.” |
| Didn’t Think About It | 15 (44.1%) - 20 (58.8%) | Did not feel a personal susceptibility to LC due to not thinking about LC prior to developing LC | “Yeah, that's something I didn't think I would deal with at all, probably. I probably had more confidence in that, that I would get COVID, get over it. And that would be the end of it.” |
| Post-Vaccine Protection | 3 (8.8%) - 6 (17.6%) | Did not feel a personal susceptibility to LC due to perception of protection after receiving the COVID vaccine prior to developing LC | “We got all of our vaccinations, we got all of the boosters. So, I did everything right and still got it.” |
| Did Not Match Media Portrayal | 0 (0.0%) - 1 (2.9%) | Did not feel a personal susceptibility to LC due to personal attributes not matching media portrayal of those typically impacted by LC prior to developing LC | “It seemed like the real risk – and the media kind of also portrayed that the real risk was to older people or people with pre-existing conditions. So, I did not anticipate at all that something might go wrong in my case.” |
| Due to Incorrect Perception | 6 (17.6%) | Did not feel a personal susceptibility to LC due to an incorrect perception of LC symptoms/durations/severity/etc. prior to developing LC | I thought that it was mainly people who were hospitalized with COVID… So those who had been hospitalized and those with comorbidities, serious comorbidities, such as immune system issues, diabetes, heart conditions, those types of things.” [Did you yourself feel susceptible to long COVID?] “No.” |
| Healthy Lifestyle | 3 (8.8%) - 7 (20.6%) | Did not feel a personal susceptibility to LC due to living a healthy lifestyle (eating/exercise/health habits) prior to developing LC | “I am usually reasonably healthy… I would get things, but never terribly seriously. And now I've got my shots, but, you know, my flu shot and all of that, but I didn't think I was overly susceptible to illness in general.” |
| **Yes** |  |  |  |
| Family History of Medical Concerns | 0 (0.0%) | Felt a personal susceptibility to LC prior to developing LC due to a family history of medical concerns | N/A |
| Preexisting Health Issue | 3 (8.8%) - 4 (11.8%) | Felt a personal susceptibility to LC prior to developing LC due to a preexisting/chronic health issue | “The symptoms were kind of, I had kind of heard that, you know, it seemed like it was a bit different for everyone, but I remember being kind of worried about it because my immune system's not the best.” |
| Age | 0 (0.0%) - 1 (2.9%) | Felt a personal susceptibility to LC prior to developing LC due to age | “I mean, I have a few of those underlying conditions that I mentioned, just a few that are pretty common (in), you know, middle-aged people.” |
| Essential Worker | 0 (0.0%) - 1 (2.9%) | Felt a personal susceptibility to LC prior to developing LC due to being an essential worker/exposure at the workplace | “The workers who were still out there, were kind of like, yeah, we're going to get it.” |
| **Acute COVID** |  |  |  |
| Yes | 14 (41.2%) - 18 (52.9%) | Did feel a personal susceptibility to COVID-19 | “You know, I guess I felt susceptible to COVID.” |
| No | 7 (20.6%) | Did not feel a personal susceptibility to COVID-19 | “No, I wasn't. I mean we took the precautions and right when vaccinations were available to the mainstream public that's right when I got it.” |
| **Other Worries** |  |  |  |
| Susceptible Others | 5 (14.7%) - 6 (17.6%) | Had concern for/perceived family members/other individuals close to them as susceptible to LC/COVID-19 | “So I had three kiddos, but I did have that worry that they would develop long COVID if we did not take precautions.” |
| Acute Complications/Death | 3 (8.8%) - 4 (11.8%) | Had concern for acute complications (hospitalization/death/etc.) associated with COVID-19 | “So yeah, it was pretty much just worried about dying from it and being on a ventilator for months and months and months.” |
