# Supplementary material for: The lived experience of long COVID: A thematic analysis of an in-depth interview study
Source: PLOS Ment Health. 2026 Feb 6;3(2):e0000500. doi: 10.1371/journal.pmen.0000500 (PMC12880701; doi:10.1371/journal.pmen.0000500)
Supplement: S4 Table — (DOCX) [file pmen.0000500.s004.docx]

**S4 Table. Daily Functioning Codes**

| **Code:** | **Code Endorsement Range:** | **Code Description:** | **Example Quotes:** |
| --- | --- | --- | --- |
| **Daily functioning** |  |  |  |
| Unchanged | 1 (2.9%) - 4 (11.8%) | Reported no change in daily functioning since developing LC | “I don't cancel social things and I'm not going to let it run my life. So I kind of just push forward with it and I'm like I'm just going to deal with it.” |
| **Changed** |  |  |  |
| Changes in eating habits | 9 (26.5%) - 10 (29.4%) | Reported changes in eating habits since developing LC | “My diet has changed completely because my stomach cannot handle certain things.” |
| Quality of life impaired | 11 (32.4%) - 13 (38.2%) | Reported changes in quality of life since developing LC | “Yeah, I don't know what it's like to be happy. I don't know what it's like to be not sick. I don't know what it's like.” |
| Plan around health | 19 (55.9%) - 21 (61.8%) | Reported increased need for planning around health/health concerns since developing LC | “I have to plan my days now… I can't, yeah, just having to basically work around my illness, like it's a part of my daily life and something that I have to incorporate with everything and it really did change almost everything…” |
| Increased time managing health | 8 (23.5%) - 9 (26.5%) | Reported increased time spent managing health/health concerns since developing LC | “I spend all my time researching a cure and trying treatments to try to get myself better.” |
| Less social interaction | 13 (38.2%) - 14 (41.2%) | Reported decreased social interactions since developing LC | “I can go days without talking to anybody.” |
| Increased online interactions | 3 (8.8%) - 5 (14.7%) | Reported increased online interactions (use of social media/online social interactions/etc.) since developing LC | “I'm going to admit, with the pandemic and everyone in my lab group leaving, most of my friends are online.” |
| House/bedbound | 14 (41.2%) - 16 (47.1%) | Reported being largely housebound or bedbound since developing LC | “Yeah, it pretty much left me housebound, if not bedbound for years.” |
| Limits daily tasks | 23 (67.6%) - 24 (70.6%) | Reported limits in time for daily tasks/ability to complete daily tasks since developing LC | “Well, I mean, it can probably cut my available hours of the day in half.” |
| Need assistance from others for daily tasks | 8 (23.5%) - 9 (26.5%) | Reported a need for assistance from others in order to complete daily tasks since developing LC | “So I'm waiting for someone that I hired to help me with stuff because it's just too much to ask my parents constantly to come over and help me unpack my suitcase so that my things can be put away.” |
