# Supplementary material for: The lived experience of long COVID: A thematic analysis of an in-depth interview study
Source: PLOS Ment Health. 2026 Feb 6;3(2):e0000500. doi: 10.1371/journal.pmen.0000500 (PMC12880701; doi:10.1371/journal.pmen.0000500)
Supplement: S5 Table — (DOCX) [file pmen.0000500.s005.docx]

**S5 Table. Extracurricular Functioning Codes**

| **Code:** | **Code Endorsement Range:** | **Code Description:** | **Example Quotes:** |
| --- | --- | --- | --- |
| **Change in Extracurricular/Career Functioning** |  |  |  |
| Unchanged | 1 (2.9%) - 5 (14.7%) | Reported no change in extracurricular or career functioning since developing LC | “I was and still currently am a preschool teacher working with all ages.” |
| **Decreased** |  |  |  |
| Household duties | 4 (11.8%) | Reported decreased involvement/ability to engagement in household duties since developing LC | “I couldn't even do laundry. Going up the stairs from doing laundry… I would have to go to bed and stay in bed for hours, sometimes days from just doing … laundry.” |
| Travel | 6 (17.6%) - 8 (23.5%) | Reported decreased involvement/ability to engagement in travel since developing LC | “I can't really like travel or, you know, I can't really have a life, really.” |
| Family events | 3 (8.8%) - 7 (20.6%) | Reported decreased involvement/ability to engagement in family events since developing LC | “I couldn't go to my son, my other son, he graduated.” |
| Community events | 1 92.9%) - 2 (5.9%) | Reported decreased involvement/ability to engagement in community events since developing LC | “And I was kind of considered a leader in that community, even though I'm new. Even though I'm new, they were asking me to be in a leadership position. And not only could I not be in a leadership position, I couldn't even participate, which is devastating for me.” |
| Social events/interactions | 10 (29.4%) - 11 (32.4%) | Reported decreased involvement/ability to engagement in social events/social interactions since developing LC | “And so I've had to stop doing things or have chosen to stop doing certain things, especially a lot of after-hours social stuff.” |
| **School** |  |  |  |
| Decreased school engagement | 1 (2.9%) - 3 (8.8%) | Reported decreased involvement/ability to engagement in school requirements/activities since developing LC | “Well, I was supposed to have graduated by now, so there's that, and I haven't even done my prelim or my final yet.” |
| Break from school | 0 (0.0%) - 2 (5.9%) | Reported taking a break/leave from school/academics since developing LC | “I'm now taking the summer off for the first time since I started undergrad basically, because, you know, it's like, okay, I need to try and get, at least figure out how to manage stuff enough to work around.” |
| **Work** |  |  |  |
| Promotion impacted | 2 (5.9%) - 3 (8.8%) | Reported negative impacts to potential/upcoming/suspected promotions since developing LC | “It probably, from a work occupational standpoint, it probably affects promotions or projects or that type of thing.” |
| Symptoms impact work | 11 (32.4%) - 13 (38.2%) | Reported LC symptoms to impact functioning in the workplace (work requirements/engagement/project completion/etc.) | “… If I have a lot of meetings late in the afternoon, I don't like those because I have a harder time staying on task.” |
| Need time off | 9 (26.5%) - 11 (32.4%) | Reported needing time off/leave from work since developing LC | “I've missed more work than I have in the past, taken more sick days.” |
| Need frequent breaks | 2 (5.9%) - 4 (11.8%) | Reported needing frequent breaks in the workplace since developing LC | “I do still have to take breaks at work.” |
| Switch jobs | 1 (2.9%) - 2 (5.9%) | Reported switching jobs since developing LC | “So I've had, after my second bout with COVID, that's when I moved to (a different job) because I couldn't do nights anymore.” |
| Switch to remote | 2 (5.9%) - 3 (8.8%) | Reported moving to remote/virtual completely/partially work since developing LC | “I did go back to work in December, but by the end of February… I started having the post-exertion and falling down and all that, I switched to remote.” |
| Switch to part time | 3 (8.8%) - 5 (14.7%) | Reported moving to part-time work, from full-time work, since developing LC | “Now I have switched to working part-time.” |
| Could not engage in project/aspect of work due to health | 9 (26.5%) - 13 (38.2%) | Reported inability to engage in aspects of job requirements/project requirements in the workplace since developing LC | “I had to adjust how I was doing my job rather than being more hands on and working with the physical part of the job. I had to kind of bury myself in more of the administrative tasks of my position.” |
| Quit | 3 (8.8%) - 6 (17.6%) | Reported quitting job since developing LC | “I stopped working (in) 2021. I can't work anymore.” |
| Lost job | 2 (5.9%) - 5 (14.7%) | Reported losing job/getting fired since developing LC | “I lost my license because I can't work. I haven't had income since 2021.” |
| **Increased** |  |  |  |
| New hobbies | 7 (20.6%) - 10 (29.4%) | Reported increased involvement in hobbies/extracurricular activities since developing LC | “So I've picked up other hobbies, you know, things like that… all solitary things.” |
| Family roles | 2 (5.9%) | Reported increased family roles/family obligations since developing LC | “But anyway, so I switched from working to being a full-time caregiver and a teacher and home school, all that good stuff…” |
