# Supplementary material for: The lived experience of long COVID: A thematic analysis of an in-depth interview study
Source: PLOS Ment Health. 2026 Feb 6;3(2):e0000500. doi: 10.1371/journal.pmen.0000500 (PMC12880701; doi:10.1371/journal.pmen.0000500)
Supplement: S6 Table — (DOCX) [file pmen.0000500.s006.docx]

**S6 Table. Health Before COVID Codes**

| **Code:** | **Code Endorsement Range:** | **Code Description:** | **Example Quotes:** |
| --- | --- | --- | --- |
| **Health Before COVID** |  |  |  |
| Mostly Healthy | 24 (70.6%) - 28 (82.4%) | Primarily in good health and/or experiencing no chronic health conditions prior to developing LC | “I'd say my health was really pretty good. I had migraines, but they didn't affect my life to any great extent. I really was pretty healthy.” |
| Had Chronic Conditions | 19 (55.9%) | Experiencing chronic health conditions and/or poor health prior to developing LC | “I had 3 medical diagnoses prior to this.” |
