# Supplementary material for: The lived experience of long COVID: A thematic analysis of an in-depth interview study
Source: PLOS Ment Health. 2026 Feb 6;3(2):e0000500. doi: 10.1371/journal.pmen.0000500 (PMC12880701; doi:10.1371/journal.pmen.0000500)
Supplement: S7 Table — (DOCX) [file pmen.0000500.s007.docx]

**S7 Table. Healthcare Interactions Codes**

| **Code:** | **Code Endorsement Range:** | **Code Description:** | **Example Quotes:** |
| --- | --- | --- | --- |
| **Healthcare Interactions** |  |  |  |
| **Recommendations for providers** |  |  |  |
| Interdisciplinary communication | 2 (5.9%) - 4 (11.8%) | Recommendation for medical providers to improve interdisciplinary communication with other providers for improved treatment of LC | “And if the cardiologist could talk to the interventional radiologist and they could talk to the rheumatologist, you know, like maybe if they all got together and talked about it, maybe they'd be like, oh, hey, this is what's going on.” |
| Exposure to people with LC | 7 (20.6%) - 11 (32.4%) | Recommendation for medical providers to have increased exposure to individuals with LC, for improved treatment of LC | “I think because of the rural and maybe a lack of exposure to a lot of people that have had it, maybe they don't know what to do with it.” |
| Timely appointments | 0 (0.0%) - 1 (2.9%) | Recommendation for medical providers to have timely appointments/limit wait times for improved treatment of LC |  |
| Simplified communication | 0 (0.0%) - 3 (8.8%) | Recommendation for medical providers to have simplified communication with patients/more effective communication with patients for improved treatment of LC | “… less scientific wording and… a way that they could understand it and (be) like, oh, okay.” |
| Appropriate documentation | 1 (2.9%) | Recommendation for medical providers to have more appropriate/accurate documentation of LC symptoms /LC illness for improved treatment of LC | “…. you may not agree with the patient, but at least write down what they tell you.” |
| Identify LC earlier | 1 (2.9%) - 6 (17.6%) | Recommendation for medical providers to more effectively and more efficiently identify/diagnose LC for improved treatment of LC | “And I think doctors are mostly missing those cases...” |
| Updating public on LC | 4 (11.8%) - 7 (20.6%) | Recommendation for medical providers to update the public on LC protocol, treatment, and identification | “If there could be more information available to the general public, it might help.” |
| Protocol/treatment development | 6 (17.6%) - 7 (20.6%) | Recommendation for medical providers to develop LC protocol and treatment | “And I wish, I mean, if there are doctors out there who are doing more specific things or might have more breakthroughs, I wish they would research them and refer more.” |
| Willingness to try alternative treatments | 2 (5.9%) | Recommendation for medical providers to increase openness/willingness to try alternative treatments in management/treatment of LC symptoms | “They're not willing to stick their neck out to try different things.” |
| Increasing knowledge of LC | 18 (52.9%) - 20 (58.8%) | Recommendation for medical providers to improve their understanding and increased their knowledge of LC symptoms and LC illness | “I think it's common enough these days that I feel like doctors should be more like informed on it...” |
| Did not seek care | 1 (2.9%) - 5 (14.7%) | Did not seek care for LC illness or LC symptoms | “I really haven't gotten any medical advice or medical recommendations at all about dealing with it.” |
| Seeing many providers | 6 (17.6%) - 7 (20.6%) | Reported being seen by multiple medical providers for treatment of LC/LC symptoms | “Um, I've seen, I've seen a lot of other specialists.” |
| **Exercise** |  |  |  |
| Did not comment | 10 (29.4%) | Reported medical providers did not comment on exercise as potential medical recommendation for LC symptoms | (Have any providers told you not to exercise with long COVID or just not really talked about exercise in relation to long COVID at all?)  “No, no providers have told me that.” |
| Discouraged | 3 (8.8%) - 4 (11.8%) | Reported medical providers to discourage exercise for patients with LC symptoms | “But they're more inclined to walk and don't get your heart rate up because it'll tire you out more. Like they're, they're the people who really want me to pace myself through it all.” |
| Encouraged | 10 (29.4%) - 12 (35.3%) | Reported medical providers to recommend and encourage exercise for patients with LC symptoms | “Like, I needed to run to get it all out. I tried that and it just made it worse.” |
| **Provider recommendations** |  |  |  |
| No recommendations | 2 (5.9%) | Reported medical providers gave little/no recommendations for treatment of LC symptoms | “They did all the tests. They don't have a clue what it was...” |
| Vague instructions (water, exercise, etc.) | 5 (14.7%) - 7 (20.6%) | Reported medical providers gave vague recommendations (water, exercise, general health, etc.) for treatment of LC symptoms | “He's like, oh, you need rest and nutrition and hydration. Okay. Well, you could use that statement for anybody for anything...” |
| Treat symptoms | 1 (2.9%) - 4 (11.8%) | Reported medical providers gave recommendations to treat specific symptoms for treatment of LC symptoms | “So attack the stomach problems first and then go to the next thing. And I think it's been really helpful and it's really nice.” |
| Specific treatments | 1 (2.9%) | Reported medical providers gave recommendations of specific treatments for treatment of LC symptoms | “And so, they just told me to get like support hose.” |
| Conflicting recommendations | 1 (2.9%) - 2 (5.9%) | Reported medical providers gave conflicting recommendations in treatment of LC symptoms | “Yeah, it has been. Oh, no, not by specialists, just by normal doctors. A doctor.” |
| Wait it out | 5 (14.7%) | Reported medical providers gave recommendations wait out symptoms/wait for improvement in symptoms in treatment of LC symptoms | “… his best recommendation was like, well then take it easy...” |
| **Attributions** |  |  |  |
| Other physical health conditions | 3 (8.8%) - 6 (17.6%) | Reported medical providers to attribute LC symptoms to other physical health conditions | “But the next thing was, oh, okay, it's not depression. Well, it must be your diabetes.” |
| Age | 3 (8.8%) | Reported medical providers to attribute LC symptoms to age | “… most (of) the doctors you see at my age say ‘at your age’ it's that little caveat they give you.” |
| Mental health | 7 (20.6%) - 8 (23.5%) | Reported medical providers to attribute LC symptoms to mental health concerns/illness | “Um, my main thing was like the doctors blaming everything on anxiety and stress and stuff.” |
| **Positive interactions** |  |  |  |
| Collaboration with patient | 5 (14.7%) - 8 (23.5%) | Reported medical providers collaboration with patients in treatment of LC, as a positive interaction with medical providers | “No, I think they did a very good job of kind of listening to me, kind of listening to what my symptoms were and kind of responding appropriately and taking the necessary action to kind of rule out different things and stuff like that, so I feel like they did a very good job.” |
| Belief in sx/LC | 8 (23.5%) - 11 (32.4%) | Reported medical providers validation of or belief in LC symptoms and/or LC illness as a positive interaction with medical providers | “It's been very good. That's why I think I've increased in my health. There's people actually listening to my symptoms and wanting to work with me.” |
| Trying new treatments | 4 (11.8%) - 6 (17.6%) | Reported medical providers trying novel treatments in treatment of LC as a positive interaction with medical providers | “And the doctor here, the pain doctor… he is doing some cutting edge research on long COVID… that has had surprisingly good results.” |
| Long COVID education | 7 (20.6%) - 11 (32.4%) | Reported medical providers education in LC symptoms/LC illness as a positive interaction with medical providers | “But I'm very lucky to have a primary (care provider) who said that sounds like long COVID.” |
| Taking time to research options | 4 (11.8%) - 6 (17.6%) | Reported medical providers engagement in research of LC/LC treatment options as a positive interaction with medical providers | “He was pretty good about it. He actually read some studies and does some video conferencing and stuff with doctors that are dealing with it now.” |
| **Negative interactions** |  |  |  |
| No answers | 18 (52.9%) - 21 (61.8%) | Reported medical providers having little/no answers about treatment of LC as a negative interaction with medical providers | “So I wouldn't say it's been great, like they don't really care, but I think I've never really had so many doctors say, ‘I don't know what to tell you.’” |
| Mischaracterizing LC | 9 (26.5%) - 11 (32.4%) | Reported medical providers mischaracterizing LC symptoms as another illness and/or giving an incorrect diagnosis of LC symptoms as a negative interaction with medical providers | “But my response was, well, I've never been diagnosed with anxiety, but I am currently quite anxious that I'm being misdiagnosed.” |
| Treating body systems as separate | 5 (14.7%) - 7 (20.6%) | Reported medical providers treating body systems as separate when treating symptoms as opposed to taking a more holistic approach, as a negative interaction with medical providers | “They've (sent me to) about a bajillion different specialists at this point, so my head's kind of spinning.” |
| Treatments/referrals that don't work | 11 (32.4%) - 15 (44.1%) | Reported medical providers recommending treatments or referrals that prove ineffective as a negative interaction with medical providers | “So in 2020, and they kind of just said that I needed to clear out my lungs. Okay. Like, I needed to run to get it all out. I tried that and it just made it worse. Okay. Okay. And then I've also been told, like, anxiety, so I need to go run to get my anxiety out as well. But, but that also didn't work.” |
| Lack of knowledge | 21 (61.8%) - 22 (64.7%) | Reported medical providers lacking knowledge/education or being unwilling to learn about LC/LC symptoms as a negative interaction with medical providers | “We'll see what we can do, but I don't know what to do for you… She really kind of blew me off.” |
| Distrust | 19 (55.9%) - 22 (64.7%) | Reported medical providers distrusting/denying patient report of symptoms, gaslighting, dismissing symptoms and/or insisting diagnostics are inconclusive/normal as a negative interaction with medical providers | “Doctors don't believe me.” |
| Increased preparation for healthcare visits | 4 (11.8%) | Reported need for increased preparation for health visits generally and/or due to previous negative experiences with medical providers | “So, I have made sure that I've got information relevant to what I think might be going on printed out with me in a folder when I go for an appointment.” |
| **Healthcare system barriers** |  |  |  |
| Wait times/wait lists | 8 (23.5%) - 9 (26.5%) | Reported wait lists and/or wait times for health appointments as a healthcare system barrier | “… the other thing is just how frustrating it is to get appointments and how far out they're scheduled with everybody.” |
| Expense | 8 (23.5%) - 11 (32.4%) | Reported cost for health appointments as a healthcare system barrier | “She stopped taking insurance and it's five hundred dollars an hour… for the first hour of a visit and then four hundred dollars for every hour after that. And when you don't work and you have zero income, you really can't help yourself.” |
| Location | 0 (0.0%) - 5 (14.7%) | Reported location as a healthcare system barrier due to lack of services in specific locations | “I live in a very small rural town and I don't know that they have a lot of background in it. I mean, they're general practitioners.” |
| Insurance company issues | 5 (14.7%) - 7 (20.6%) | Reported insurance company difficulties regarding health appointments as a healthcare system barrier | “The thing that came to mind was insurance stop making calls, because the health care providers actually make the decisions and make the calls.” |
| Disability insurance | 3 (8.8%) - 5 (14.7%) | Reported difficulty of obtaining disability insurance for LC and/or lack of disability insurance for LC as a healthcare system barrier | “I was denied the first two times and now I have a hearing.  It changed a little bit after, like, social security, they never recognized long COVID as a natural disability in the beginning.” |
| Alternative medicine accessibility | 3 (8.8%) - 4 (11.8%) | Reported difficulty in accessing alternative medicine treatments and providers as a healthcare system barrier | “And if there is, they're all holistic or naturopathic and you pay out of pocket. That's the only one that I have seen. And right now they're charging (a lot) for long COVID.” |
| Other | 4 (11.8%) - 8 (23.5%) | Reported other healthcare system barriers | “You know, for example, it's really hard for them to research anything treatment wise that isn't a pill, right? Because, you know, big pharma really backs that and pays for that.” |
