# Supplementary material for: The lived experience of long COVID: A thematic analysis of an in-depth interview study
Source: PLOS Ment Health. 2026 Feb 6;3(2):e0000500. doi: 10.1371/journal.pmen.0000500 (PMC12880701; doi:10.1371/journal.pmen.0000500)
Supplement: S8 Table — (DOCX) [file pmen.0000500.s008.docx]

**S8 Table. Long COVID Provider Codes**

| **Code:** | **Code Endorsement Range:** | **Code Description:** | **Example Quotes:** |
| --- | --- | --- | --- |
| **Healthcare interactions** |  |  |  |
| **Providers seen for LC** |  |  |  |
| Primary care provider | 20 (58.8%) - 21 (61.8%) | Reported seeing a primary care provider for the treatment/diagnosis/management of LC or symptoms/diagnoses comorbid with LC | “In general, you know, I get a physical once a year and I've talked to my doctor about it at that time.” |
| GI doctor | 1 (2.9%) - 3 (8.8%) | Reported seeing a GI physician for the treatment/diagnosis/management of LC or symptoms/diagnoses comorbid with LC | “I go to a gastroenterologist” |
| Pain medicine/anesthesiology | 1 (2.9%) | Reported seeing a pain medicine or anesthesiologist for the treatment/diagnosis/management of LC or symptoms/diagnoses comorbid with LC | “And then referred me at that point to a pain doctor … who is doing cutting edge research on long-term COVID and pain relief.” |
| Neurologist | 7 (20.6%) - 8 (23.5%) | Reported seeing a neurologist for the treatment/diagnosis/management of LC or symptoms/diagnoses comorbid with LC | “I go to a … neurologist…” |
| Nephrologist | 2 (5.9%) - 3 (8.8%) | Reported seeing a nephrologist for the treatment/diagnosis/management of LC or symptoms/diagnoses comorbid with LC | “I have a nephrology appointment...” |
| Rheumatologist | 5 (14.7%) | Reported seeing a rheumatologist for the treatment/diagnosis/management of LC or symptoms/diagnoses comorbid with LC | “I have a rheumatologist.” |
| Long COVID specialist | 5 (14.7%) - 6 (17.6%) | Reported seeing a Long COVID Specialist for the treatment/diagnosis/management of LC or symptoms/diagnoses comorbid with LC | “And so it wasn't until December of 2022 when I got into long COVID specialists where I have like a good doctor who actually listened and actually heard my symptoms for long COVID rather than, Oh, this person is sick and they might have long COVID, but most doctors didn't acknowledge that I had it.” |
| Pulmonologist | 6 (17.6%) | Reported seeing a pulmonologist for the treatment/diagnosis/management of LC or symptoms/diagnoses comorbid with LC | “I go to a …. pulmonologist…” |
| Endocrinologist | 1 (2.9%) | Reported seeing an endocrinologist for the treatment/diagnosis/management of LC or symptoms/diagnoses comorbid with LC | “When I got down here, the GP decided to send me to the neurologist, to the rheumatologist, to the endocrinologist first.” |
| Cardiologist | 8 (23.5%) - 9 (26.5%) | Reported seeing a cardiologist for the treatment/diagnosis/management of LC or symptoms/diagnoses comorbid with LC | “I go to a… cardiologist” |
| Infectious disease | 1 (2.9%) | Reported seeing an infectious disease physician for the treatment/diagnosis/management of LC or symptoms/diagnoses comorbid with LC | “Yeah, he sent me to, like, infectious disease and did blood work and told me I was like, fine.” |
| Hematologist/Oncologist | 1 (2.9%) - 2 (5.9%) | Reported seeing a hematologist/oncologist for the treatment/diagnosis/management of LC or symptoms/diagnoses comorbid with LC | “I saw a number of specialists, I saw a blood doctor, hematologist, and they thought I might have a cancer…” |
| Allergist | 2 (5.9%) | Reported seeing an allergist for the treatment/diagnosis/management of LC or symptoms/diagnoses comorbid with LC | “I ended up seeing an allergist” |
| Dermatologist | 1 (2.9%) | Reported seeing a dermatologist for the treatment/diagnosis/management of LC or symptoms/diagnoses comorbid with LC | “So then I went on to see a dermatologist.” |
| ENT | 2 (5.9%) | Reported seeing an ENT for the treatment/diagnosis/management of LC or symptoms/diagnoses comorbid with LC | “I saw the, um, ENT to check my ears to see if that was part of the problem.” |
| Internal medicine | 1 (2.9%) | Reported seeing an internal medicine physician for the treatment/diagnosis/management of LC or symptoms/diagnoses comorbid with LC | “I've seen an internal medicine doctor” |
| Osteopathic | 1 (2.9%) | Reported seeing an osteopathic physician for the treatment/diagnosis/management of LC or symptoms/diagnoses comorbid with LC | “It took, I would say, a good five years for me to find a DO, an osteopathic doctor.” |
| Chiropractor | 3 (8.8%) | Reported seeing a chiropractor for the treatment/diagnosis/management of LC or symptoms/diagnoses comorbid with LC | “I was also referred by the pain doc, not just physical therapy but also the chiropractor and that helped a lot as well.” |
| Physical therapy | 7 (20.6%) - 8 (23.5%) | Reported seeing a physical therapist for the treatment/diagnosis/management of LC or symptoms/diagnoses comorbid with LC | “I was also referred by the pain doc, not just physical therapy but also the chiropractor and that helped a lot as well.” |
| Occupational therapy | 2 (5.9%) | Reported seeing an occupational therapist for the treatment/diagnosis/management of LC or symptoms/diagnoses comorbid with LC | “I've been in occupational therapy for about a year and a half.” |
| Speech therapy | 0 (0.0%) - 3 (8.8%) | Reported seeing a speech therapist for the treatment/diagnosis/management of LC or symptoms/diagnoses comorbid with LC | “The neurologist sent me for vestibular therapy and for cognitive speech therapy.” |
| Functional medicine provider | 3 (8.8%) | Reported seeing a functional medicine provider for the treatment/diagnosis/management of LC or symptoms/diagnoses comorbid with LC | “I'm actually, I just started seeing a functional medicine doctor who is happy to look at some of the different things and test different things than… regular doctors are doing.” |
| Acupuncture | 0 (0.0%) - 2 (5.9%) | Reported seeing an acupuncturist for the treatment/diagnosis/management of LC or symptoms/diagnoses comorbid with LC | “I've done acupuncture.” |
| Other | 11 (32.4%) - 12 (35.3%) | Reported seeing another type of provider for the treatment/diagnosis/management of LC or symptoms/diagnoses comorbid with LC | “Um, I've been to, I go to a gastroenterologis, pulmologist, cardiologist, neurologist, urologist, and I think I'm missing one.” |
