# Supplementary material for: The lived experience of long COVID: A thematic analysis of an in-depth interview study
Source: PLOS Ment Health. 2026 Feb 6;3(2):e0000500. doi: 10.1371/journal.pmen.0000500 (PMC12880701; doi:10.1371/journal.pmen.0000500)
Supplement: S9 Table — (DOCX) [file pmen.0000500.s009.docx]

**S9 Table. Long COVID Theories Codes**

| **Code:** | **Code Endorsement Range:** | **Code Description:** | **Example Quotes:** |
| --- | --- | --- | --- |
| **LC theories** |  |  |  |
| No theory | 6 (17.6%) - 8 (23.5%) | Reported no theory/rationale regarding personal development of LC | “There's all the conspiracy theories, but I don't know.” |
| Previous life stress | 1 (2.9%) - 5 (14.7%) | Reported previous life stress as theory/rationale for personal development of LC | “We always have life stuff going on… there's always a lot going on.” |
| Pre-COVID health | 11 (32.4%) - 17 (50.0%) | Reported pre-COVID health/previous health conditions as theory/rationale for personal development of LC | “I don't know whether it's just because of the, you know, being older and the fact that I do have the issues with bronchitis that I was just more susceptible…” |
| Genetics | 7 (20.6%) | Reported genetics/genetic make-up as theory/rationale for personal development of LC | “…Now (they are) coming out with a lot of stuff about genetics and long COVID...” |
| Chance | 4 (11.8%) - 5 (14.7%) | Reported chance/luck as theory/rationale for personal development of LC | “Chance maybe.” |
| Doing too much during/following initial infection | 5 (14.7%) - 7 (20.6%) | Reported excessive activity level as theory/rationale for personal development of LC | “But the severity of my second one, I attribute to having to physically go back to being very, very physically active afterwards.” |
| Many COVID infections | 1 (2.9%) | Reported high number of COVID infections as theory/rationale for personal development of LC | “I think having it that many times, that's what my doctor said is that she said, well of course you've had it four times you're going to get long COVID or something like that.” |
| Age | 4 (11.8%) - 7 (20.6%) | Reported age as theory/rationale for personal development of LC | “A lot of it seems to be women in their 50s and 60s who didn't necessarily have a really bad case of it, but (have) not been able to shake it.” |
| COVID never left body | 3 (8.8%) - 4 (11.8%) | Reported idea that COVID remained inside the body after initial infection as theory/rationale for personal development of LC | “Yeah, it's more like I wonder if I have a viral load somewhere, you know, a pocket of infection, you know, somewhere that there's a residual or I'm in the overreaction phase.” |
| Severity of initial infection | 2 (5.9%) - 3 (8.8%) | Reported severity of initial COVID infection as theory/rationale for personal development of LC | “Well, I've always kind of thought of it in terms of it's just the damage that was done when I had COVID.” |
| Mental health | 0 (0.0%) - 2 (5.9%) | Reported current/previous mental health as a contributing factor to development LC | “I knew something was off, but I didn't know if it was mental health.” |
| Other medical theories | 3 (8.8%) | Reported certain medical theories as a contributing factor to development LC | “From what I just read, and it gets into the cells, and if it's in the cells of your brain, it's got to do something to your brain.” |
| Lack of physical activity | 1 (2.9%) | Reported lack of exercise as a contributing factor to development LC | “You know, I do have a suspicion that if I was more intentional in moving my body and stretching and exercising it, there might be some improvement, at least in the muscle soreness part.” |
