# Supplementary material for: The lived experience of long COVID: A thematic analysis of an in-depth interview study
Source: PLOS Ment Health. 2026 Feb 6;3(2):e0000500. doi: 10.1371/journal.pmen.0000500 (PMC12880701; doi:10.1371/journal.pmen.0000500)
Supplement: S10 Table — (DOCX) [file pmen.0000500.s010.docx]

**S10 Table. Long COVID Trajectory Codes**

| **Code:** | **Code Endorsement Range:** | **Code Description:** | **Example Quotes:** |
| --- | --- | --- | --- |
| **Long COVID trajectory** |  |  |  |
| **Personal LC** |  |  |  |
| Unsure | 10 (29.4%) - 12 (25.3%) | Reported uncertainty in trajectory of personal LC symptoms/illness over time and/or reported uncertainty in future trajectory of LC symptoms | “I mean, I know there are a lot of people with long COVID who… have gotten like better. So I have hope that it'll either get better or (I will) be better at adapting to it.” |
| Unchanged | 15 (44.1%) - 17 (50.0%) | Reported no change personal LC symptoms/illness over time and/or reported prediction of no change in LC symptoms in the future | “And I've kind of given up hope in most of my symptoms that trouble me (improving). So my prognosis is learning to live with this condition and being more accepting of it.” |
| Worsening | 15 (44.1%) | Reported worsened personal LC symptoms/illness over time and/or reported prediction of worsening in LC symptoms in the future | And then about a year later, I realized I wasn't getting better and I was starting to get worse again when it came to fatigue, needing a lot more sleep. |
| Improving | 25 (73.5%) - 26 (76.5%) | Reported improvement in personal LC symptoms/illness over time and/or reported prediction of improvement in LC symptoms in the future | Neurologically, the neurological stuff… I’ve definitely gotten better since the first summer. |
| **Long COVID, in general** |  |  |  |
| Need more research | 19 (55.9%) - 20 (58.8%) | Expressed opinion that more research is needed about LC as an illness | But there, of course, needs to be more information out there. There needs to be better accessibility to it. (There) needs to be more in-depth research on a lot of different areas. |
| Need medical cure | 9 (26.5%) - 10 (29.4%) | Expressed opinion that LC will need medical professional intervention/a cure | I think without a good medical cure, I don't think I'm going to cure naturally. |
