# Supplementary material for: The lived experience of long COVID: A thematic analysis of an in-depth interview study
Source: PLOS Ment Health. 2026 Feb 6;3(2):e0000500. doi: 10.1371/journal.pmen.0000500 (PMC12880701; doi:10.1371/journal.pmen.0000500)
Supplement: S11 Table — (DOCX) [file pmen.0000500.s011.docx]

**S11 Table. Mental Health Codes**

| **Code:** | **Code Endorsement Range:** | **Code Description:** | **Example Quotes:** |
| --- | --- | --- | --- |
| **Mental health changes** |  |  |  |
| **Prior mental health** |  |  |  |
| MH symptoms/conditions | 7 (20.6%) - 11 (32.4%) | Reported experiencing previous mental health symptoms/conditions prior to developing LC | “I have dealt with depression and anxiety and ADHD.” |
| No prior MH symptoms | 21 (61.8%) | Denied experiencing previous mental health symptoms/conditions prior to developing LC | “I feel like, I don't know, it's like my anxiety is out of control.  I never had this stuff before.” |
| **MH Treatments** |  |  |  |
| Did not seek MH care | 0 (0.0%) - 2 (5.9%) | Denied seeking mental health care due to LC | (Have you sought medication or counseling for your mental health since having COVID?)  “No.” |
| **MH care** |  |  |  |
| Medication | 11 (32.4%) - 13 (38.2%) | Received/sought out medication to manage mental health symptoms/conditions developed as a result of LC | “Since I got COVID, I've started seeing somebody for my mental health and I've started medication for my mental health.” |
| Counseling/MH techniques | 15 (44.1%) - 17 (50.0%) | Received/sought out counseling or other mental health techniques to manage mental health symptoms/conditions developed as a result of LC | “I mean, I think it helps kind of just… process through different emotions and different thoughts that I might have and just kind of work through those.” |
| Depends on day | 0 (0.0%) | Reported that mental health symptoms vary day to day since developing LC | N/A |
| Unchanged | 4 (11.8%) - 8 (23.5%) | Reported no change in mental health symptoms since developing LC | “I think, like I said before, the biggest ongoing issue I have is the taste and smell stuff, but I do my best to not let it get me down or not to become too focused on why me or why this is happening.” |
| Improved | 1 (2.9%) - 4 (11.8%) | Reported improved mental health since developing LC | “I'd say it probably makes me less anxious because I'm… interacting with the world less.” |
| **Worsened** |  |  |  |
| Stress | 8 (23.5%) - 11 (32.4%) | Reported new or worsened stress since developing LC | “… maybe stressing more about things that I can't control...” |
| Dissociation/Disconnected | 2 (5.9%) | Reported new or worsened dissociation/disconnection since developing LC | “And I get that depersonalization quite a lot, which sort of just makes me feel... like I'm not myself.” |
| Emotionally numb | 0 (0.0%) - 1 (2.9%) | Reported feeling emotionally numb since developing LC | “It's just that it's really hard … to feel strongly emotionally about something if you are so fatigued that you're kind of numb.” |
| Self-harm/suicidal ideation | 7 (20.6%) | Reported self-harm behaviors and/or suicidal ideation since developing LC | “I don't want to stick around.” |
| Paranoia | 1 (2.9%) | Reported new or worsened mental health symptoms since developing LC | “Like I would just become inconsolable and convinced that everyone was out to get me, like completely paranoid of everybody, including my own children.” |
| Less able to hold emotions inside | 1 (2.9%) | Reported decreased ability to manage emotions since developing LC | “I'm less able to keep stuff inside.” |
| Discouraged | 6 (17.6%) - 12 (35.3%) | Reported new or worsened feeling of discouragement since developing LC | “I think I've become more discouraged.” |
| Irritability | 1 (2.9%) | Reported new or worsened feeling of irritability since developing LC | “I'm more irritable.” |
| Embarrassed | 1 (2.9%) - 4 (11.8%) | Reported new or worsened feeling of embarrassment since developing LC | “And it's like so humiliating.” |
| Trapped | 1 (2.9%) - 5 (14.7%) | Reported new or worsened feeling of being trapped since developing LC | “And that I was stuck essentially in this body… nowhere to go and nothing, no cure, no nothing.” |
| Lack of control/autonomy | 9 (26.5%) - 13 (38.2%) | Reported feeling new or worsened lack of control since developing LC | “I feel like I have less like autonomy.” |
| Self-blame | 3 (8.8%) - 6 (17.6%) | Reported new or worsened feeling of self-blame since developing LC | “I'm very frustrated with myself that I can't push through this, or that I can't make myself deal with this in a better manner.” |
| Hopeless | 3 (8.8%) - 10 (29.5%) | Reported new or worsened feeling of hopelessness since developing LC | “After almost three years of this, I kind of do give up hope.” |
| One thing after another/relentless | 5 (14.7%) - 6 (17.6%) | Reported new or worsened feeling of relentlessness of personal situation since developing LC | “I have dealt with one thing … then it cools off for a little bit and then another thing will come by or like another illness or symptom or something that I'll have to deal with again.” |
| Self-conscious | 1 (2.9%) - 2 (5.9%) | Reported new or worsened feeling of self-consciousness since developing LC | “Yeah, because, you know, I've gained weight and I just don't feel as good.” |
| Uncertainty | 27 (79.4%) - 28 (82.4%) | Reported new or worsened feeling of uncertainty since developing LC | “Cause I don't know if I'm like still going to have this, you know?” |
| Denial | 1 (2.9%) | Reported new or worsened feeling of denial since developing LC | “Every single time a crash happens after doing completely normal things. I'm just in disbelief about it. Disbelief, angst, denial.” |
| Loneliness/isolation | 8 (23.5%) - 12 (35.3%) | Reported new or worsened feeling of isolation/loneliness since developing LC | “I don't fit in anywhere. I don't feel like I fit in anywhere. Nobody believes me. There's not a single person who believes me.” |
| Grief | 23 (67.6%) - 24 (70.8%) | Reported new or worsened feeling of grief since developing LC | “I lost a huge thing that I didn't already grieve because, oh, I lost, like, the ability to do all these experiences before.” |
| Crying, weeping | 2 (5.9%) - 6 (17.6%) | Reported increased crying/weeping since developing LC | “I've never cried as much as I've cried in the last little bit.” |
| Sadness | 10 (29.4%) - 13 (38.2%) | Reported new or worsened feeling of sadness since developing LC | “I mean, like, I'm sad that I'm not able to go out and do things.” |
| Anger | 8 (23.5%) - 13 (38.2%) | Reported new or worsened feeling of anger since developing LC | “Just like anger at not being able to do something that I feel like I should be able to do, um, something that was easy before.” |
| Depression | 18 (52.9%) | Reported new or worsened feeling of depression since developing LC | “Depression is here 24-7. It doesn't matter how I'm feeling. I feel depressed.” |
| Apathy | 2 (5.9%) - 5 (14.7%) | Reported new or worsened feeling of apathy since developing LC | “You know, the sleep is not good sleep and just, you know… kind of the apathetic attitude.” |
| Frustration | 21 (61.8%) - 27 (79.4%) | Reported new or worsened feeling of frustration since developing LC | “But it's frustrating because I want to go out and do things.” |
| **Anxiety/panic** |  |  |  |
| Trajectory of long COVID | 11 (32.4%) | Reported new or worsened feeling of anxiety/panic regarding the trajectory of LC since developing LC | “So I do worry about new symptoms or worse symptoms down the road.” |
| Career worries | 0 (0.0%) - 1 (2.9%) | Reported new or worsened feeling of anxiety/panic regarding their career since developing LC | “So it's more of trying to find something that I can do that will also not stress me out so bad in that I can actually have like a fluctuating schedule if I get sick really badly to kind of prepare for the future.” |
| Cognitive worries | 3 (8.8%) - 4 (11.8%) | Reported new or worsened feeling of anxiety/panic regarding cognitive deficits since developing LC | “… that brain fog… concerns me the most I would say.” |
| Illness/COVID susceptibility | 16 (47.1%) - 17 (50.0%) | Reported new or worsened feeling of anxiety/panic regarding illness and/or COVID susceptibility since developing LC | “Other than that, I don't have much issues besides the anxiety of like being around a lot of people just like just because I'm sick and more susceptible to everything.” |
| Finances | 0 (0.0%) - 3 (8.8%) | Reported new or worsened feeling of anxiety/panic regarding finances since developing LC | “We are suffering financially.” |
| Unclear/other | 16 (47.1%) - 17 (50.0%) | Reported other/unclear new or worsened feelings of anxiety/panic since developing LC | “The symptoms definitely, my bad days, my anxiety gets so bad, and it gets the worst of me.” |
| Guilt | 3 (8.8%) - 6 (17.6%) | Reported new or worsened feeling of guilt since developing LC | “I feel, you know, guilty because (I'm) kind of not, you know, able to provide for myself and I'm sucking my mom's (savings) dry.” |
| Shame | 2 (5.9%) - 3 (8.8%) | Reported new or worsened feeling of shame since developing LC | “It takes a lot to get over the shame of needing help and asking for help.” |
| Post-traumatic stress disorder (PTSD) /Trauma | 3 (8.8%) - 4 (11.8%) | Reported new or worsened PTSD/trauma since developing LC | “I was diagnosed with PTSD and I have agoraphobia.” |
| **Acceptance** |  |  |  |
| Reports acceptance | 14 (41.2%) | Indicated acceptance of current life situation | “Yeah, I think I'm accustomed to knowing that it's not going to be like it was before.” |
| Reports lack of acceptance | 2 (5.9%) - 6 (17.6%) | Indicated lack of acceptance of current life situation | “I don't want to accept that this could be my normal.” |
| No energy for MH symptoms | 3 (8.8%) - 4 (11.8%) | Reported lack of energy to manage mental health symptoms | “I just don't have the energy to be that anxious.” |
| MH symptoms due to LC | 3 (8.8%) - 4 (11.8%) | Reported development of mental health symptoms since developing LC | (Do you want to (seek to improve) the physical symptoms first? Those would be your goal?)  “Yeah. Actually, once those are addressed, there'd be nothing to be depressed about.” |
