# Supplementary material for: The lived experience of long COVID: A thematic analysis of an in-depth interview study
Source: PLOS Ment Health. 2026 Feb 6;3(2):e0000500. doi: 10.1371/journal.pmen.0000500 (PMC12880701; doi:10.1371/journal.pmen.0000500)
Supplement: S12 Table — (DOCX) [file pmen.0000500.s012.docx]

**S12 Table. Perceived Social Support Codes**

| **Code:** | **Code Endorsement Range:** | **Code Description:** | **Example Quotes:** |
| --- | --- | --- | --- |
| **Perceived social support** |  |  |  |
| **Social media** |  |  |  |
| **Recommendation** |  |  |  |
| LC awareness | 0 (0.0%) - 2 (5.9%) | Recommendation for increased awareness of LC on social media/discussions about LC in the media to improve support for those with LC | “Yes, I think more education. I think more attention, even in the media.” |
| Satisfactory | 2 (5.9%) - 5 (14.7%) | Reported satisfaction with support received through social media interaction | “I have a very good Twitter long COVID group.” |
| Unsatisfactory | 0 (0.0%) | Reported dissatisfaction with support received through social media interaction |  |
| **LC support group** |  |  |  |
| Satisfactory | 2 (5.9%) | Reported satisfaction with support received through LC support groups | “I have …. a care group on Facebook, and additionally at (school), we have a group of physicians, nurses, and mid-level providers who all got long COVID, and we communicate.” |
| Unsatisfactory | 1 (2.9%) - 2 (5.9%) | Reported dissatisfaction with support received through LC support groups | “I learned that you can't join … groups with long COVID because most of those people, and great for them, have never had COVID, so they don't understand.” |
| **Others/Society** |  |  |  |
| Satisfactory | 1 (2.9%) - 2 (5.9%) | Reported satisfaction with support received from others/society | “And some that are truly like sympathetic and empathetic and what can I do to help, blah, blah, blah.” |
| **Unsatisfactory** |  |  |  |
| Milder symptoms | 2 (5.9%) - 3 (8.8%) | Reported dissatisfaction with support received from others/society due to experiencing milder symptoms that expected by others | “I think like I say, the big thing is that people with the milder symptoms, I think can get neglected and looked over.” |
| **Visibility** |  |  |  |
| Wish LC were more visible | 9 (26.5%) - 11 (32.4%) | Described desire for LC symptoms to be more visible due to dissatisfaction with support from others/society | “… a lot of my symptoms aren't, they're not visible … I worry that it seems like I'm just whining.” |
| Wish LC were less visible | 1 (2.9%) - 3 (8.8%) | Described desire for LC symptoms to be less visible due to dissatisfaction with support from others/society | “And I just hate that that's how I come off is this old dude who can't breathe or this guy who stumbles as he walks.” |
| **Judgement** |  |  |  |
| Long COVID | 3 (8.8%) - 6 (17.6%) | Described feeling judgement from others due to developing LC | “And the C word is so controversial … I knew no one was going to believe me. I knew people were going to tell me that it was from the vaccination, because that's what most people believe that the vaccination was giving you … COVID.” |
| Safety precautions (mask, etc.) | 5 (14.7%) - 8 (23.5%) | Described feeling judgement from others due to taking safety precautions (wearing a mask/etc.) | “But like my husband, he doesn't want to go to places even masked … because he's scared that someone's going to harass us because so many people have been getting harassed from wearing masks.” |
| Lack of knowledge | 2 (5.9%) - 4 (11.8%) | Reported dissatisfaction with support received from others/society due to their lack of knowledge about LC | “I think most people really just don't understand how ill I am, probably because I look well and they don't get it.” |
| Criticism/Blame | 0 (0.0%) - 3 (8.8%) | Reported dissatisfaction with support received from others/society due to experiencing criticism/blame from others | “And I got lectured several times about how dumb I was being.” |
| Incorrect attribution | 0 (0.0%) - 1 (2.9%) | Reported dissatisfaction with support received from others/society due to experiencing others making incorrect attributions about their symptoms/impacts of LC | “I knew people were going to tell me that it was from the vaccination, because that's what most people believe that the vaccination was giving you on COVID.” |
| Disbelief | 7 (20.6%) - 9 (26.5%) | Reported dissatisfaction with support received from others/society due to experiencing disbelief in LC from others | “Nobody believes me. There's not a single person who believes me.” |
| **Employers/Educators** |  |  |  |
| **Suggestions** |  |  |  |
| Support groups for LC | 1 (2.9%) | Recommendation for support group availability in academia/workplace for individuals with LC | “I think one thing for larger companies is maybe getting a support group for those that (have long COVID).” |
| Improved awareness of LC | 5 (14.7%) - 7 (20.6%) | Recommendation for improved awareness of LC/LC knowledge in academia/workplace | “… educate yourself on the variety of ways that long COVID manifests … believe their employees when they say they're not feeling well, to provide adequate time for them to heal, to provide insurance that will cover the cost of whatever treatment is needed.” |
| Accommodations for long COVID | 8 (23.5%) - 11 (32.4%) | Recommendation for accommodation availability in academia/workplace for individuals with LC | “And I would say I wish schools could know more about it and could take more accommodations into account.” |
| Long COVID as a disability | 2 (5.9%) - 4 (11.8%) | Recommendation for recognition of LC as a disability in academia/workplace | “Like, I don't think (insurance company) was going to give me my disability unless (employer) put pressure on them and I bet you a lot of them are that way and just having that process sort of easier and streamlined (would be helpful).” |
| **Unsatisfactory** |  |  |  |
| No information about COVID positives | 0 (0.0%) - 2 (5.9%) | Reported dissatisfaction with employer/educator lack of knowledge regarding LC positive tests |  |
| Expectation to return to prior level | 5 (14.7%) - 7 (20.6%) | Reported dissatisfaction with employer/educator expectation to return to prior level of functioning with LC | “I might need a little more time. And they were like, you have to come back. You've been out for too long.” |
| Lack of understanding | 6 (17.6%) - 7 (20.6%) | Reported dissatisfaction with employer/educator lack of understanding/knowledge of LC | “Yeah, they were not very understanding … they tried to … coerce or like threaten me to come back to work.” |
| Encouragement to resign or quit | 1 (2.9%)- 2 (5.9%) | Reported dissatisfaction with employer/educator encouragement to resign or quit due to experience with LC | “Um, and they tried to, like, I don't know, coerce or like threaten me to come back to work. And I was also told that I needed to like resign or quit.” |
| **Satisfactory** |  |  |  |
| Accommodations/Flexibility | 9 (26.5%) | Reported satisfaction with available accommodations/flexibility in academia/workplace around experience of LC | “Yeah, when I had the headache, I was able to request a … blue light and glare guard for my computer screen.” |
| Understanding | 11 (32.4%) - 13 (38.2%) | Reported satisfaction with level of understanding in academia/workplace around experience of LC | “Really pretty supportive. My boss has been very supportive. Um, colleagues have been supportive.” |
| **Friends/Family** |  |  |  |
| **Suggestion** |  |  |  |
| Offering assistance | 3 (8.8%) - 4 (11.8%) | Recommendation for family/friends to offer assistance (physically/emotionally) to those with LC | “And if somebody would just say, like, tell me what you want me to do for the day and I could say, hang a picture here, like, wipe up that stain over there. Hey, move this rock here.” |
| Sympathy | 4 (11.8%) | Recommendation for family/friends to have sympathy for those with LC | “Take a minute, have some sympathy.” |
| Respecting Limits | 3 (8.8%) - 5 (14.7%) | Recommendation for family/friends to respect the limits (physically/emotionally/mentally/etc.) of those with LC | “I've had a lot of people, I mean, I've had my friends adapt their plans around me to make sure that I'm not missing out on things that I really appreciate.” |
| Improving education/understanding | 8 (23.5%) - 10 (29.4%) | Recommendation for family/friends to improve their education/understanding of LC | “So I think the primary piece of advice I would give a family member is to believe them when they say they don't feel well and give them permission to heal in whatever way their body needs to heal, whether that's resting or going for short walks ...” |
| **Unsatisfactory** |  |  |  |
| Pity | 2 (5.9%) - 3 (8.8%) | Reported dissatisfaction with experiencing pity from friends/family due to experiencing LC | “I think like from everyone I knew before … they're more pitying and generally less helpful.” |
| Incorrect attribution | 1 (2.9%) - 5 (14.7%) | Reported dissatisfaction with friends/family incorrectly attributing LC illness/symptoms | “And then, and then there's days that like my mom will be like, ‘Oh, honey, that's not from long COVID. You're getting older. Like that's from getting older.’” |
| Placing blame | 5 (14.7%) - 10 (29.4%) | Reported dissatisfaction with friends/family placing blame around developing of LC | “I mean, I had an aunt who was like, you just want … somebody to pay attention to you.” |
| "All in your head" | 3 (8.8%) - 4 (11.8%) | Reported dissatisfaction with friends/family assertion that LC/LC symptoms are “all in your head” or due to a mental concern | “And my stepmom insinuated that I needed like mental health medication and brought up a history of like family mental illness and I just thought … am I losing it?” |
| Misguided advice | 7 (20.6%) - 9 (26.5%) | Reported dissatisfaction with friends/family giving wrong/misguided/unhelpful advice regarding LC | “And I have a lot of family members who read a lot … oh, well, have you considered this? ...” |
| Not understanding limits | 10 (29.4%) | Reported dissatisfaction with friends/family not understanding functioning (physical/mental/emotion) limits due to LC | “No one makes accommodations. If I'm not feeling good, but something needs to get done, I have to do it. A lot of people forget it.” |
| Disbelief | 16 (47.1%) - 18 (52.9%) | Reported dissatisfaction with disbelief in LC/LC symptoms from friends/family | “And I don't think they realize the extent cause I'd just be on the phone telling them I'm sleeping all the time and (they were) not … actually like believing it, I guess.” |
| **Satisfactory** |  |  |  |
| Adapting activities | 3 (8.8%) | Reported satisfaction with friends/family adapting activities/being understanding of functioning limits with LC | “Um, especially my sister lives with me, which has been great. So like when we go grocery shopping, if it's a day that I'm feeling dizzy, I'm the one that drives the cart.” |
| Emotional support | 18 (52.9%) - 19 (55.9%) | Reported satisfaction with emotional support from friends/family regarding LC | “Pretty much everyone I've met since has been super like understanding and helpful.” |
| Belief in sx | 15 (44.1%) - 16 (47.1%) | Reported satisfaction with friends/family belief in LC symptoms and/or LC as an illness | “Um, I don't think anybody has not believed me. They know what I was like before.” |
| Willingness to educate self | 3 (8.8%) - 6 (17.6%) | Reported satisfaction with friends/family willingness to educate themselves on LC/LC symptoms and impact | “It's kind of fun when they're interested and I can talk about what I've read and learned ...” |
| Patience | 1 (2.9%) - 2 (5.9%) | Reported satisfaction with friends/family patience with impacts of LC/LC symptoms | “Well, I would say people have been very patient with my way of being.” |
| Physical assistance | 6 (17.6%) - 7 (20.6%) | Reported satisfaction with physical assistance from friends/family due to LC symptoms | “He gets me food that I need, he's willing to, you know, if I'm sitting on the couch with my leg up and heating pads all around me, he will go get me something to drink.” |
| Avoiding polarizing conversations | 2 (5.9%) - 4 (11.8%) | Reported satisfaction with friends/family willingness to avoid polarizing conversations about LC | “We decided not to discuss those matters.” |
