# Supplementary material for: The lived experience of long COVID: A thematic analysis of an in-depth interview study
Source: PLOS Ment Health. 2026 Feb 6;3(2):e0000500. doi: 10.1371/journal.pmen.0000500 (PMC12880701; doi:10.1371/journal.pmen.0000500)
Supplement: S13 Table — (DOCX) [file pmen.0000500.s013.docx]

**S13 Table. Perception of Self Codes**

| **Code:** | **Code Endorsement Range:** | **Code Description:** | **Example Quotes:** |
| --- | --- | --- | --- |
| **Perception of self** |  |  |  |
| Unchanged | 13 (38.2%) - 19 (55.9%) | Reported unchanged perception of self/personality due to LC | “Yeah, it's, you know, other than it's slowed me down a little bit, I'm pretty much the same person.” |
| **Changed** |  |  |  |
| **Positive** |  |  |  |
| Increased awareness | 1 (2.9%) - 7 (20.6%) | Reported personal perception of increased awareness since developing LC | “… there's a bit more consideration… try to stay a bit more conscientious of what's going on with me…” |
| More motivation to fight | 3 (8.8%) - 6 (17.6%) | Reported personal perception of increased motivation of fight since developing LC | “I'm really motivated to not be in the state that I'm in.” |
| Discovered gender identity | 1 (2.9%) | Reported discovering gender identity since developing LC | “I realized I was trans early on in becoming ill.” |
| Proud | 1 (2.9%) - 3 (8.8%) | Reported personal perception of increased pride since developing LC | “I'm actually much prouder of myself than I would have been before because I'm like, oh, that's why I felt the way I felt for so long.” |
| Less serious | 0 (0.0%) - 1 (2.9%) | Reported personal perception of decreased seriousness since developing LC | “I can sit with it and not get super upset about it like I used to.” |
| Deeper | 1 (2.9%) - 2 (5.9%) | Reported personal perception of increased depth as an individual since developing LC | “But I think I am a little deeper than I was before.” |
| More sensitive | 2 (5.9%) | Reported personal perception of increased sensitivity since developing LC | “I think I'm way more sensitive and a better person in that sense.” |
| More empathetic/compassionate | 11 (32.4%) - 12 (35.3%) | Reported personal perception of increased empathy and compassion since developing LC | “… it's given me a lot of compassion for people with chronic illness.” |
| New ability to stand up for self | 1 (2.9%) | Reported new ability to advocate for self since developing LC | “It kind of has forced me to believe in myself more, like before I was still learning how to really kind of like stand up for myself.” |
| Less fear of dying | 1 (2.9%) | Reported decreased fear of death since developing LC | “I never really feared death, but I feared dying. And my experience kind of took that away, because if I'm in the hospital and I go through anything similar to what I went through then, I don't think I have that same fear.” |
| Better social ties | 0 (0.0%) - 3 (8.8%) | Reported improved social relationships since developing LC | “But definitely, those relationships have strengthened tremendously my family, my best friends, my new support friends, and such.” |
| More flexible | 2 (5.9%) | Reported personal perception of increased flexibility since developing LC | “So perhaps a little more flexible than I would have been...” |
| **Negative** |  |  |  |
| More anxious | 0 (0.0%) - 3 (8.8%) | Reported perception of self as more anxious since developing LC | “… there have probably been more anxious moments just in general, not related to COVID so much, worrying about being sick, but more in maybe stressing more about things that I can't control…” |
| Edgy | 1 (2.9%) | Reported perception of self as edgy since developing LC | “Well, you know… I feel myself edgy this morning.” |
| Bitter | 1 (2.9%) - 2 (5.9%) | Reported perception of self as bitter since developing LC | “Kind of frustrated, definitely frustrated and kind of bitter.” |
| Worthless | 2 (5.9%) | Reported perception of self as worthless since developing LC | “I feel like I'm a worthless human.” |
| Jealous | 1 (2.9%) | Reported perception of self as jealous since developing LC | “And then I (am) jealous that all these people are like, oh yeah, I was fine a week later.” |
| Less optimistic | 1 (2.9%) - 2 (5.9%) | Reported perception of self as less optimistic since developing LC | “Certainly you don't, I'm not going to say I'm depressed, but I don't have quite as good of an outlook as I had.” |
| Less forgiving | 0 (0.0%) - 2 (5.9%) | Reported perception of self as less forgiving since developing LC | “I think that I'm a lot harsher on myself. Like, I guess when I can't do something, okay, I'm less forgiving of myself.” |
| Harsher on self | 1 (2.9%) - 3 (8.8%) | Reported perception of self as harsher on self since developing LC | “… I get really frustrated and that can lead to kind of anger at myself and then just sadness.” |
| Less capable | 2 (5.9%) - 6 (17.6%) | Reported perception of self as less capable since developing LC | “I don't feel like I'm as capable in terms of doing all the tasks in my job as I was before.” |
| General dislike for current self | 1 (2.9%) | Reported general dislike for current self since developing LC | “Yeah… I don't like this person.” |
| Dependent | 0 (0.0%) - 4 (11.8%) | Reported perception of self as dependent since developing LC | “Well, I'm dependent on someone else to do my ADLs (activities of daily living) now.” |
| More serious | 2 (5.9%) - 4 (11.8%) | Reported perception of self as more serious since developing LC | “It not not hugely different, like, you know, but as I said before, maybe a little bit more serious, maybe a little bit more acknowledgment of the fact that, you know, yeah, your days aren't granted to you necessarily...” |
| Less ambitious | 2 (5.9%) - 4 (11.8%) | Reported perception of self as less ambitious since developing LC | “Uh, I think I've become less ambitious.” |
| Less intellectual | 0 (0.0%) - 1 (2.9%) | Reported perception of self as less intellectual since developing LC | “I used to feel I could hold my own in conversations, in discussions, in intellectual pursuits, and I don't feel like I can do that anymore.” |
| More self-conscious | 1 (2.9%) - 2 (5.9%) | Reported perception of self as more self-conscious since developing LC | “Or I'm self-conscious of how they're perceiving me.” |
| More Dependent | 4 (11.8%) - 5 (14.7%) | Reported perception of self as more dependent since developing LC | “So I'm used to being very independent and having to rely on somebody to do everything for me is hard.” |
| Decreased ability to handle adversity | 0 (0.0%) - 4 (11.8%) | Reported decreased ability to manage adversity since developing LC | “I feel like I would have been able to handle all of the family stress better if I had more energy and if it wasn't for COVID.” |
| Less patient | 4 (11.8%) | Reported perception of self as less patient since developing LC | “Oh, I'm a lot less patient with people who do what I consider stupid things.” |
| Less sharp/slower | 1 (2.9%) - 4 (11.8%) | Reported perception of self as less sharp and/or slower since developing LC | “The brain fog thing is really bothersome because I know I'm not as sharp as I was.” |
| Older | 2 (5.9%) - 3 (8.8%) | Reported perception of self as older since developing LC | “I feel older. I feel like I went from kind of vibrant to old.” |
| Less confident | 1 (2.9%) - 2 (5.9%) | Reported perception of self as less confident since developing LC | “But I think that I was more happy-go-lucky, and I was, I (felt a) lot more confident, I was more happy-go-lucky, and I feel (I took) a lot more for granted.” |
| Less happy | 2 (5.9%) - 4 (11.8%) | Reported perception of self as less happy since developing LC | “But I do think, I mean, I think I'd be happier if I didn't have it.” |
| **Resonate less with identities** |  |  |  |
| Spouse | 0 (0.0%) - 2 (5.9%) | Reported resonating less with spousal identity since developing LC | “It's so opposite to who I am, both my work identity and my social identity, my identity within my family.” |
| Parent | 0 (0.0%) - 3 (8.8%) | Reported resonating less with parental identity since developing LC | “I don't, you know, when they're going through tough times, I don't really have energy to support them in the way that I would have in the past.” |
| Work | 1 (2.9%) - 6 (17.6%) | Reported resonating less with work/career identity since developing LC | “It's a valuable thing, obviously, but like my self-worth is completely separate now from the ability to be productive.” |
| Less competent | 2 (5.9%) - 3 (8.8%) | Reported perception of self as less competent since developing LC | “… I feel completely not competent. Not competent to do the things I could with work. Not as competent doing the things I need to do as a mother or as a wife.” |
| Less social | 9 (26.5%) - 11 (32.4%) | Reported perception of self as less social since developing LC | “I don't feel comfortable talking to people and carrying on conversations.” |
| Subdued | 2 (5.9%) - 4 (11.8%) | Reported perception of self as subdued since developing LC | “I'm more subdued than I used to be.” |
| Depersonalization/not self anymore | 10 (26.5%) | Reported depersonalization and/or significant change in perception of self since developing LC | “Like, I don't feel things the way I used to. And I get that depersonalization quite a lot, which sort of just makes me feel like just like I'm not myself.” |
| Dulled | 2 (5.9%) - 3 (8.8%) | Reported perception of self as dulled since developing LC | “But like personality-wise, I mean, it has very much dulled me.” |
| Boring | 1 (2.9%) - 3 (8.8%) | Reported perception of self as boring since developing LC | “I was a completely different person and I feel like I'm boring now.” |
| Vulnerable | 4 (11.8%) - 9 (26.5%) | Reported perception of self as vulnerable since developing LC | “Maybe a little bit more vulnerable.” |
