# Supplementary material for: The lived experience of long COVID: A thematic analysis of an in-depth interview study
Source: PLOS Ment Health. 2026 Feb 6;3(2):e0000500. doi: 10.1371/journal.pmen.0000500 (PMC12880701; doi:10.1371/journal.pmen.0000500)
Supplement: S14 Table — (DOCX) [file pmen.0000500.s014.docx]

**S14 Table. Perception of Self Codes**

| **Code:** | **Code Endorsement Range:** | **Code Description:** | **Example Quotes:** |
| --- | --- | --- | --- |
| **Perspective Changes** |  |  |  |
| **Unchanged** |  |  |  |
| Views of others | 13 (38.2%) - 14 (41.2%) | Reported unchanged perspective/view of others after developing LC | “You know, I don't (view) other people differently.” |
| Nothing has changed as a result of COVID/LC | 0 (0.0%) | Reported unchanged perspectives as a result of LC/COVID |  |
| Worldview | 13 (38.2%) - 15 (44.1%) | Reported unchanged worldview after developing LC | (Would you say your worldview has been affected by your experience?)  “No, I wouldn't.” |
| Life purpose | 17 (58.8%) | Reported unchanged life purpose after developing LC | (Did your purpose of life change?)  “Not purpose of life.” |
| Values | 16 (47.1%) - 20 (58.8%) | Reported unchanged values after developing LC | “I'm still you know, I have the same value(s), you know, the same person.” |
| Importance of support system (family, friends) | 1 (2.9%) - 5 (14.7%) | Reported unchanged perspective of the importance of family after developing LC | “… the support of my family is so good. They're, I feel like I have them no matter what.” |
| Health system is broken | 0 (0.0%) - 2 (5.9%) | Reported unchanged perspective that the health system is broken after developing LC | “… the world isn't really set up for disabled people.” |
| **Changed** |  |  |  |
| **Society/Others** |  |  |  |
| Less tolerance | 13 (38.2%) - 14 (41.2%) | Reported less tolerance for society/others after developing LC | “I could surround myself with people who had all the same beliefs and I can't anymore...” |
| Increased hope | 1 (2.9%) - 2 (5.9%) | Reported increased hope in society/others after developing LC | “So I don't, when I look at the future, I don't see a return to the status quo as I had it in 2019, but I am mildly hopeful that with the studies of treatments that are coming out and more research being paid to it, that there will be more treatments available to manage symptoms and that there's more awareness of it now.” |
| Decreased hope | 11 (32.4%) - 13 (38.2%) | Reported decreased hope in society/others after developing LC | “It's a very scary thing, I don't think I've had a pendulum swing in my lifetime and I'm worried about what that means for my grandchildren and I'm worried about the number of people who can, who are so willing to follow politicians who are making decisions that are not in their best interest.” |
| Flexibility | 0 (0.0%) - 1 (2.9%) | Noted change in perception of the flexibility of society/others after developing LC | “And I was like, oh, well, we can adjust to this.” |
| Politics | 20 (58.8%) | Reported change in feelings/thoughts/opinions around politics after developing LC | “… it makes me angry and I've never been this angry… or discouraged about our whole political system as I am now and that became so apparent during COVID.” |
| Dismissal of COVID | 15 (44.1%) - 16 (47.1%) | Reported change in perspective of those who dismiss COVID after developing LC | “If I get mad at anything, I get mad at people who refuse to get the vaccine when it first became available which allowed the virus to mutate and get worse and worse and more prevalent until now when it's endemic.” |
| Less in-person interaction | 1 (2.9%) - 2 (5.9%) | Reported perspective of less in-person interaction in society/between individuals after developing LC | “Because people started relying more on social media and computers than actual interaction with people.” |
| More social media | 3 (8.8%) - 4 (11.8%) | Reported perspective of more social media interaction in society/between individuals after developing LC | “I think people had time to sit at home and live on social media and just absorb all the propaganda.” |
| Distrust in government | 5 (14.7%) - 7 (20.6%) | Reported distrust in the government after developing LC | “We can't believe our government… who, where do we go? And I think my brain's broken from that. So I don't know what to say or do anymore. And I think it's… really, really hard.” |
| **Personal** |  |  |  |
| Better understanding of self | 2 (5.9%) - 4 (11.8%) | Reported improved understanding of self after developing LC | “Yeah, I have had to do so much soul searching just to be okay with being sick that I'm a much different person.” |
| Flexibility | 0 (0.0%) | Reported changed perception of personal flexibility after developing LC | N/A |
| Advocacy | 5 (14.7%) | Reported changes perception of advocacy (personal/other) after developing LC | “I feel like I should advocate for myself more sometimes to get more coverage or more care or more belief that this is, that what I'm going through is impacting things.” |
| Reflection/Appreciation | 13 (38.2%) | Reported changes in perception of personal reflection abilities and appreciation of self after developing LC | “It does help your outlook a lot when you can get back to the things you liked.” |
| **Worldview** |  |  |  |
| Not fair | 1 (2.9%) - 2 (5.9%) | Reported perception of world as unfair after developing LC | “I think my belief on the fairness of that notion has changed.” |
| Optimistic | 3 (8.8%) - 7 (20.6%) | Reported optimism in perception of the world after developing LC | “I try to keep a really positive outlook, I look for the good things, I contribute in the ways I can contribute.” |
| Focusing more on self | 5 (14.7%) | Reported increased focus on self after developing LC | “I'm trying to do a lot more self-care now than I used to. I've found that it's become more necessary.” |
| Taking less for granted | 6 (17.6%) - 7 (20.6%) | Reported increased appreciation/taking less for granted after developing LC | “It's just more about coping and living in the now and being grateful for what I've got.” |
| Being more present | 2 (5.9%) - 6 (17.6%) | Reported being more present after developing LC | “You know that life is short. These things can come on. Something can happen. Don't worry about it. Don't worry about finances. I'm trying to, trying to let a lot of things go.” |
| Enjoy things more | 3 (8.8%) - 6 (17.6%) | Reported enjoying things/events/activities/life more after developing LC | “I, I think I'm stressing now on, um, trying to enjoy things more.” |
| Pessimistic increase | 11 (32.4%) - 13 (38.2%) | Reported increased pessimism in perception of the world after developing LC | “Yeah, I just saw that like life is a lot harder and not everything's like sunshine and rainbows.” |
| **Personal Relationships** |  |  |  |
| Recognize more superficiality | 4 (11.8%) - 7 (20.6%) | Reported recognizing more superficiality in personal relationships after developing LC | “…we can talk or, you know, let me know how you're feeling. And then when you do, they, they don't bother.” |
| Lost trust | 2 (5.9%) - 6 (17.6%) | Reported losing trust in personal relationships after developing LC | “I don't trust people that much anymore.” |
| **Morals/values** |  |  |  |
| Changed | 6 (17.6%) - 10 (29.4%) | Reported change in morals/values after developing LC | “I changed a lot of my morals. I had to switch my whole entire life around and kind of just look at a whole new perspective of life, I guess.” |
| Weakened | 2 (5.9%) - 3 (8.8%) | Reported morals/values to have weakened after developing LC | “Like, I stood strong on my, my beliefs and my values and stuff like that. Now I question.” |
| Strengthened | 0 (0.0%) - 6 (17.6%) | Reported morals/values to have strengthened after developing LC | “Yes, it's reinforced some of my thinking, my beliefs.” |
| Life Purpose | 7 (20.6%) - 10 (29.4%) | Reported changed life purpose after developing LC | “I've always been very purpose driven, but I often feel lack of that now.” |
| **Medicine** |  |  |  |
| Reduced trust in medical field | 12 (35.3%) | Reported reduced trust in the medical field after developing LC | “We definitely need more healthcare providers who believe in COVID in the first place. So I haven't done much… to reach out to the medical community to help me at all.” |
| Change to alternative medicine | 4 (11.8%) | Reported utilization of alternative medicine after developing LC | “So it's given me more respect for any medicine that's not, you know, just Western medicine.” |
| Health System is Broken | 1 (2.9%) - 6 (17.6%) | Reported developing perspective that the health system is broken after developing LC | “… our health care system is extremely broken.” |
