# Supplementary material for: The lived experience of long COVID: A thematic analysis of an in-depth interview study
Source: PLOS Ment Health. 2026 Feb 6;3(2):e0000500. doi: 10.1371/journal.pmen.0000500 (PMC12880701; doi:10.1371/journal.pmen.0000500)
Supplement: S15 Table — (DOCX) [file pmen.0000500.s015.docx]

**S15 Table. Perception of Self Codes**

| **Code:** | **Code Endorsement Range:** | **Code Description:** | **Example Quotes:** |
| --- | --- | --- | --- |
| **Physical activity change** |  |  |  |
| **Type of physical activity** |  |  |  |
| Aerobic activity | 20 (58.8%) - 23 (67.6%) | Engaged in aerobic activity as physical activity with LC. | “And the weird thing is, I can ride my bike, and I can take my dog for a walk.” |
| Activities of daily living | 10 (29.4%) - 15 (44.1%) | Engaged in activities of daily living as physical activity with LC. | “It’s just, you know, day to day activity getting from point A to point B.” |
| Weightlifting | 4 (11.8%) | Engaged in weightlifting as physical activity with LC. | “No, I'm just going to start doing some weightlifting. I started up a couple of weeks ago, but that's pretty much stationary, just, um, handheld weights.” |
| Physical therapy | 5 (14.7%) - 7 (20.6%) | Engaged in physical therapy as physical activity with LC. | “I'm still doing physical therapy three days a week.” |
| Wants to exercise more | 12 (35.3%) - 14 (41.2%) | Expressed a desire to engage in more exercise at the time of the interview. | “I wish I could be exercising more.” |
| Finding limits/pacing | 21 (61.8%) - 26 (76.5%) | Described the process/learning to/success/lack of success with finding limits and pacing exercise or physical activity due to LC symptoms. | “I'm trying to learn how to pace my energy that I still have, but it's still a long road...” |
| **Change in physical activity level** |  |  |  |
| Unchanged | 2 (5.9%) - 4 (11.8%) | Described no change in physical activity level due to LC. | “And there's really been no change since after that. It's still that same as long as I'm feeling like that same kind of level of activity.” |
| Decreased | 27 (79.4%) - 28 (82.4%) | Described decreased engagement in physical activity level due to LC. | “Like, I can't walk any distances. I don't do anything.” |
| Increased | 0 (0.0%) - 1 (2.9%) | Described increased engagement in physical activity level due to LC. | “I don't take in a lot of calories, and now I exercise a bunch.” |
| **Experience with exercise** |  |  |  |
| Improved physical health | 4 (11.8%) - 5 (14.7%) | Described improved physical health after engagement in physical exercise. | “Now there have also been a couple of times where it's like, okay, the symptoms are present, I go on running, it makes it better...” |
| Improved mental/cognitive health | 11 (32.4%) - 12 (35.3%) | Described improved mental/cognitive health after engagement in physical exercise. | “I feel like there's times where it's like I've had a bad day at work and I go for a run, it kind of helps me to clear my mind and kind of put me in a better mental state...” |
| **Worsened physical health** |  |  |  |
| Chest pain | 3 (8.8%) | Described experiencing chest pain during/after engagement in physical exercise. | “And I also bought like a reclined exercise bike and I would bike even though I had this really bad chest pain.” |
| Heart rate issues | 9 (26.5%) - 10 (29.4%) | Described experiencing heart rate changes during/after engagement in physical exercise. | “I can go on a walk for about an hour, which is (what) he recommends, but I can't do anything more than that because my heart rate gets up really high.” |
| Post-exertional malaise | 19 (55.9%) - 24 (70.6%) | Described experiencing post exertional malaise during/after engagement in physical exercise. | “Sometimes, you know, if I'm having a good morning and I feel like I have energy and I go and do something, you know, sometimes by the afternoon, I'm really sorry I did, and that may last the rest of the day, or that may last for two days or whatever, so that's more of a post exertional malaise thing, but usually in the afternoon, I'm sagging.” |
| Blood pressure changes | 0 (0.0%) | Described experiencing blood pressure changes during/after engagement in physical exercise. | N/A |
| Dizziness | 2 (5.9%) - 3 (8.8%) | Described experiencing dizziness during/after engagement in physical exercise. | “.. a lot of times… my heart's racing, I'm dizzy, I can't stand, you know, I can't stay balanced...” |
| Balance issues | 1 (2.9%) - 2 (5.9%) | Described experiencing balance issues during/after engagement in physical exercise. | “But golf, I'm worried about the balance issue with golfing. And I'm worried probably more about failing and not being able to than the golf itself.” |
| Shortness of breath | 9 (26.5%) | Described experiencing shortness of breath during/after engagement in physical exercise. | “But again, exertion definitely gets me coughing again.” |
| Muscle mass/weakness | 4 (11.8%) - 6 (17.6%) | Described experiencing decreased muscle mass/weakness during/after engagement in physical exercise. | “And the thing that was very disturbing and upsetting was I had this new muscle weakness that I never had before...” |
| Tired/fatigue | 17 (50.0%) - 18 (52.9%) | Described experiencing fatigue/tiredness during/after engagement in physical exercise. | “I'm going to try to start walking in the next few weeks, but the problem is it fatigues me...” |
| Increased soreness | 2 (5.9%) | Described experiencing increased soreness during/after engagement in physical exercise. | “(When) walking, I'd get from the parking garage to the fountain and my legs would hurt, like my butt and my thighs and hamstrings.” |
| Increased symptoms, unclear which | 3 (8.8%) | Described experiencing an increase in physical LC symptoms during/after engagement in physical exercise, however, symptoms are unclear. | “Well, sometimes there's a little bit more discomfort afterwards, immediately afterwards if I overdo (exercise).” |
| **Worsened mental/cognitive health** |  |  |  |
| Increased symptoms, unclear which | 7 (20.6%) - 8 (23.5%) | Described experiencing an increase in mental/cognitive LC symptoms during/after engagement in physical exercise, however, symptoms are unclear. | “The fact that I can't exercise as much as I used to, yeah, does affect my mood.” |
| Cognitive issues/brain fog | 1 (2.9%) - 2 (5.9%) | Described experiencing an increase in cognitive issues and/or brain fog during/after engagement in physical exercise, however, symptoms are unclear. | “The only thing (exercise) really does is tire me out. I'm very tired. My brain fog is kind of more standing in that point.” |
| No changes with exercise | 9 (26.5%) - 11 (32.4%) | Described no change in physical and/or mental health symptoms during/after engagement in physical exercise. | “I don't think it impacts the symptoms at all. It's more of the symptoms impact the exercise.” |
| Unpredictable | 4 (11.8%) - 7 (20.6%) | Described unpredictability in the impact of exercise on physical and/or mental health symptoms of LC. | “Some days it can make me more tired … and then other days it can energize me and make me feel better.” |
| Physical activity depends on symptoms | 2 (5.9%) - 7 (20.6%) | Described that LC symptoms impact ability to engage in physical exercise/activity | “I don't think it impacts the symptoms at all. It's more of the symptoms impact the exercise.” |
