# Supplementary material for: The lived experience of long COVID: A thematic analysis of an in-depth interview study
Source: PLOS Ment Health. 2026 Feb 6;3(2):e0000500. doi: 10.1371/journal.pmen.0000500 (PMC12880701; doi:10.1371/journal.pmen.0000500)
Supplement: S16 Table — (DOCX) [file pmen.0000500.s016.docx]

**S16 Table. Physical Health Codes**

| **Code:** | **Code Endorsement Range:** | **Code Description:** | **Example Quotes:** |
| --- | --- | --- | --- |
| **Physical health changes** |  |  |  |
| Unchanged | 0 (0.0%) | Reported no change in physical health due to development of LC | N/A |
| Improved | 1 (2.9%) | Reported improved physical health due to development of LC | “I don't feel like I have asthma the way I used to.” |
| **Worsened function** |  |  |  |
| Loss of mobility | 2 (5.9%) - 6 (17.6%) | Reported loss of mobility due to development of LC/LC symptoms. | “My mobility is still a bit limited.” |
| Mobility device | 6 (17.6%) - 7 (20.6%) | Reported usage/necessity of a mobility device due to development of LC/LC symptoms. | “To leave the house, I'm still in a wheelchair.” |
| Lost ability to walk | 1 (2.9%) | Reported losing ability to walk due to development of LC/LC symptoms. | “I have subsequently lost my ability to walk well.” |
| Falling | 3 (8.8%) - 5 (14.7%) | Reported falling over due to development of LC/LC symptoms. | “Really bad dizziness and falling.” |
| **Continued symptoms** |  |  |  |
| Sore throat | 5 (14.7%) - 6 (17.6%) | Reported a sore throat which persisted since developing LC/over the course of experiencing LC | “Well, immediately because the throat irritation was pretty bad and it didn't let up.” |
| Hair loss | 1 (2.9%) | Reported hair loss which persisted since developing LC/over the course of experiencing LC | “I guess I think my hair was falling out and just the tiredness and not having as much energy as I had before.” |
| Congestion | 3 (8.8%) - 4 (11.8%) | Reported congestion which persisted since developing LC/over the course of experiencing LC | “I'm always a little bit congested.” |
| Increased illness susceptibility | 2 (5.9%) - 3 (8.8%) | Reported increased illness susceptibility which persisted since developing LC/over the course of experiencing LC | “I feel like I am more susceptible to getting sick.” |
| Liver issues | 2 (5.9%) - 3 (8.8%) | Reported liver issues which persisted since developing LC/over the course of experiencing LC | “And because of my elevated liver enzymes, I saw a liver doctor...” |
| Kidney issues | 3 (8.8%) | Reported kidney issues which persisted since developing LC/over the course of experiencing LC | “I've had problems with my kidneys, my liver, my everything” |
| Pancreas issues | 1 (2.9%) | Reported pancreas issues which persisted since developing LC/over the course of experiencing LC | “They did a CT… came back with kidney and pancreas stuff.” |
| Rash | 4 (11.8%) | Reported a rash which persisted since developing LC/over the course of experiencing LC | “I started getting rashes like all around my eyes and my face and my arms.” |
| Difficulty breathing/shortness of breath | 10 (29.4%) - 11 (32.4%) | Reported difficulty breathing and/or shortness of breath which persisted since developing LC/over the course of experiencing LC | “… it feels like I cannot really breathe at all.” |
| Urinary incontinence | 1 (2.9%) | Reported urinary incontinence which persisted since developing LC/over the course of experiencing LC | “Oh, urinary incontinence, I was just peeing everywhere.” |
| Vision issues | 3 (8.8%) - 4 (11.8%) | Reported vision issues which persisted since developing LC/over the course of experiencing LC | “Literally, my eyes, it almost happened overnight. They went terrible. I never had glasses prior to this.” |
| Mouth sores | 1 (2.9%) | Reported mouth sores which persisted since developing LC/over the course of experiencing LC | “But I had those sores in my mouth.” |
| Spider veins | 0 (0.0%) - 1 (2.9%) | Reported spider veins which persisted since developing LC/over the course of experiencing LC | “And I had these little capillary things that I had never seen before, like purple mini lines in my legs and feet, I guess.” |
| Poor circulation | 0 (0.0%) | Reported poor circulation which persisted since developing LC/over the course of experiencing LC | N/A |
| Hives | 1 (2.9%) - 2 (5.9%) | Reported hives which persisted since developing LC/over the course of experiencing LC | “And then I was having random places on my body where I would get hives.” |
| Mast cell activation | 1 (2.9%) - 3 (8.8%) | Reported mast cell activation which persisted since developing LC/over the course of experiencing LC | “I've been diagnosed with mast cell activation… but has been exacerbated to the point where it's undeniable.” |
| POTS/Orthostatic intolerance | 4 (11.8%) - 8 (23.5%) | Reported POTS/Orthostatic intolerance which persisted since developing LC/over the course of experiencing LC | “The orthostatic intolerance was quite bad.” |
| Hearing loss | 1 (2.9%) | Reported hearing loss which persisted since developing LC/over the course of experiencing LC | “And I lost the peripheral vision… hearing was diminished on my left side.” |
| Tremors | 4 (11.8%) - 6 (17.6%) | Reported tremors which persisted since developing LC/over the course of experiencing LC | “I have tremors in both of my legs… it's still ongoing causing disability.” |
| Bell's Palsy | 1 (2.9%) - 2 (5.9%) | Reported bell’s palsy which persisted since developing LC/over the course of experiencing LC | “I got Bell's Palsy.” |
| Impulsivity | 0 (0.0%) - 1 (2.9%) | Reported impulsivity which persisted since developing LC/over the course of experiencing LC | “I'll do my best to try to maintain my impulsivity that got like 100 times worse with COVID too.” |
| Numbness/Nerve issues | 9 (26.5%) - 11 (32.4%) | Reported numbness and/or nerve issues which persisted since developing LC/over the course of experiencing LC | “I have neuropathy in both feet as a result.” |
| Muscle/joint pain | 12 (35.3%) - 15 (44.1%) | Reported muscle/joint pain which persisted since developing LC/over the course of experiencing LC | “I've got all kinds of lower body muscle and joint pain and they haven't been able to really figure out whether it's some sort of psoriatic arthritis or if it's something else.” |
| Balance issues | 5 (14.7%) - 9 (26.5%) | Reported balance issues which persisted since developing LC/over the course of experiencing LC | “I've been getting more dizzy, lightheaded, having issues balancing a lot more.” |
| Dizziness, lightheaded, vertigo | 14 (41.2%) - 15 (44.1%) | Reported dizziness, lightheadedness, and/or vertigo which persisted since developing LC/over the course of experiencing LC | “It would come on as like dizziness and like a faintness.” |
| **Cognitive symptoms** |  |  |  |
| Memory | 11 (32.4%) - 12 (35.3%) | Reported memory difficulties which persisted since developing LC/over the course of experiencing LC | “I still have a lot of gaps, a lot of gaps in my memory...” |
| Aphasia/word loss | 9 (26.5%) - 12 (35.3%) | Reported aphasia and/or word loss which persisted since developing LC/over the course of experiencing LC | “It wasn't until probably – it might have been a whole year later where I started to realize my word recall was pretty shot.” |
| Brain fog | 16 (47.1%) - 17 (50.0%) | Reported brain fog which persisted since developing LC/over the course of experiencing LC | “… the brain fog or whatever is so bad that I forget a lot of stuff. I lose track in the middle of sentences.” |
| Other cognitive | 20 (58.8%) | Reported other cognitive concerns which persisted since developing LC/over the course of experiencing LC | “It was on the way home and literally started feeling like I was falling out of the car. Like, I was in a roller coaster and it was all neurological things.” |
| Post-exertional malaise | 21 (61.8%) - 22 (64.7%) | Reported post-exertional malaise which persisted since developing LC/over the course of experiencing LC | “… I still have the post-exertional malaise and more with mental exertion than with physical exertion, actually.” |
| Chest pain | 7 (20.6%) - 9 (26.5%) | Reported chest pain which persisted since developing LC/over the course of experiencing LC | “I have chest pains, but I did a full cardiac workup at the hospital.” |
| Chest/respiratory irritation | 2 (5.9%) - 6 (17.6%) | Reported chest/respiratory irritations which persisted since developing LC/over the course of experiencing LC | “But initially it hit in the bronchial area, like a very dry bronchitis, and then it came back and it was in the upper respiratory area.” |
| Cough | 10 (29.4%) | Reported a cough which persisted since developing LC/over the course of experiencing LC | “Well the cough has yet to go away.” |
| Ringing in ears/tinnitus | 4 (11.8%) | Reported tinnitus/ringing in the ears which persisted since developing LC/over the course of experiencing LC | “… after that I've had ringing in my ears.” |
| Loss of muscle mass | 6 (17.6%) - 7 (20.6%) | Reported loss of muscle mass which persisted since developing LC/over the course of experiencing LC | “What has been surprising to me is how quickly I lost the use of my muscle.” |
| Abdominal pain | 2 (5.9%) - 3 (8.8%) | Reported abdominal pain which persisted since developing LC/over the course of experiencing LC | “Well, like I said, I wouldn't be able to eat any food for a couple of days and then would gradually, you know, I could eat a little bit and it felt less painful.” |
| Diarrhea | 3 (8.8%) - 5 (14.7%) | Reported diarrhea which persisted since developing LC/over the course of experiencing LC | “Yeah, I had diarrhea like every day for months.” |
| Vomiting | 3 (8.8%) | Reported vomiting which persisted since developing LC/over the course of experiencing LC | “I was either vomiting or had bad indigestion.” |
| Nausea | 5 (14.7%) - 10 (29.4%) | Reported nausea which persisted since developing LC/over the course of experiencing LC | “I still get nauseous sometimes and like throw up.” |
| **Sensitivities** |  |  |  |
| Light | 6 (17.6%) | Reported sensitivity to light which persisted since developing LC/over the course of experiencing LC | “I would get really confused and it was super like sensory overwhelmed, and I had light sensitivity to the point that I like got blackout curtains and was in a dark room for … many months, probably like around six months to a year.” |
| Food | 4 (11.8%) - 5 (14.7%) | Reported sensitivity to food which persisted since developing LC/over the course of experiencing LC | “So I just stick with the, like, four things that I know I can eat. I haven't eaten a real meal in, like, two and a half years.” |
| Sound | 5 (14.7%) | Reported sensitivity to sounds which persisted since developing LC/over the course of experiencing LC | “I was noise sensitive and I still am to an extent...’ |
| Medication/drug | 2 (5.9%) | Reported sensitivity to medications/drugs which persisted since developing LC/over the course of experiencing LC | “And then strangely enough, I developed a caffeine intolerance.” |
| Smells | 1 (2.9%) - 3 (8.8%) | Reported sensitivity to smells which persisted since developing LC/over the course of experiencing LC | “I was really sensitive to light, sound, smell...” |
| Chemicals | 2 (5.9%) | Reported sensitivity to chemicals which persisted since developing LC/over the course of experiencing LC | “I am hypersensitive to chlorine now where I was not before.” |
| Air quality | 1 (2.9%) - 2 (5.9%) | Reported sensitivity to air quality which persisted since developing LC/over the course of experiencing LC | “…we're on a code red air quality day because of the forest fires from Canada, the smoke has finally hit here and I was just like, oh, I feel horrible today.” |
| Less stamina | 16 (47.1%) | Reported having less stamina which persisted since developing LC/over the course of experiencing LC | “…knowing that stamina with the fatigue that we had to take more breaks and stop and maybe not go as far.” |
| Heart rate issues/palpitations | 12 (35.3%) - 14 (41.2%) | Reported heart rate issues and/or palpitations which persisted since developing LC/over the course of experiencing LC | “So I'm still experiencing heart rate fluctuations.” |
| Blood pressure issues | 2 (5.9%) - 3 (8.8%) | Reported blood pressure issues which persisted since developing LC/over the course of experiencing LC | “I had really bad blood pressure spikes, I remember, like they would go up really high and then they would… like a flat line.” |
| **Sleep issues** |  |  |  |
| Insomnia | 5 (14.7%) - 7 (20.6%) | Reported insomnia which persisted since developing LC/over the course of experiencing LC | “Well, I have to try as hard as I can to get enough sleep, which has meant… insomnia.” |
| Other sleep issue/unclear | 7 (20.6%) - 12 (35.3%) | Reported other/unclear sleep issues which persisted since developing LC/over the course of experiencing LC | “I have a very restless sleep...” |
| Fatigue/exhaustion | 30 (88.2%) - 32 (94.1%) | Reported fatigue/exhaustion which persisted since developing LC/over the course of experiencing LC | “I guess it's like exhaustion or fatigue again and that's always there and sometimes I'm tired.” |
| Voice affected | 1 (2.9%) - 2 (5.9%) | Reported impacts to the voice which persisted since developing LC/over the course of experiencing LC | “For instance, I've had a hoarse voice ever since coming out.” |
| Headache/migraine | 14 (41.2%) - 16 (47.1%) | Reported headaches and/or migraines which persisted since developing LC/over the course of experiencing LC | “I suffer from the headaches, I think now a few times a week.” |
| Widespread pain/other pain | 9 (26.5%) - 12 (35.2%) | Reported widespread/other pain which persisted since developing LC/over the course of experiencing LC | “Sure, I still have a good deal of pain.” |
| Vascular issues | 2 (5.9%) | Reported vascular issues which persisted since developing LC/over the course of experiencing LC | “And I've got some aneurysms...” |
| Loss of hunger | 3 (8.8%) - 5 (14.7%) | Reported loss of hunger which persisted since developing LC/over the course of experiencing LC | “I couldn't eat for a while. I still barely eat.” |
| Temperature regulation issues | 3 (8.8%) - 4 (11.8%) | Reported temperature regulation concerns which persisted since developing LC/over the course of experiencing LC | “That's another thing I have trouble with now is temperature regulation.” |
| Swelling | 2 (5.9%) - 3 (8.8%) | Reported swelling which persisted since developing LC/over the course of experiencing LC | “And then the next day I'll notice swelling in my joints, knees, ankles, elbows, shoulders, and across my back and my hands.” |
| Fainting | 4 (11.8%) | Reported fainting which persisted since developing LC/over the course of experiencing LC | “And then I fainted, which for me has never happened.” |
| Phantom smell | 2 (5.9%) - 3 (8.8%) | Reported phantom smell which persisted since developing LC/over the course of experiencing LC | “Every once in a while, I still get the phantom smells…” |
| Changes in taste, smell | 9 (26.5%) - 10 (29.4%) | Reported changes in taste and/or smell which persisted since developing LC/over the course of experiencing LC | “Everything I ate tasted like smoke. My taste and smell were really, really off and they still are.” |
| Menstrual cycle changes | 1 (2.9%) | Reported menstrual cycle changes which persisted since developing LC/over the course of experiencing LC | “So my periods were actually different than they were before because they were getting lighter, just less everything, no cramps, no anything. And then I got COVID and my next two periods were horrific, like just awful.” |
| Weight changes | 1 (2.9%) - 2 (5.9%) | Reported weight changes which persisted since developing LC/over the course of experiencing LC | “I lost (weight), so I'm getting some weight back.” |
