# Supplementary material for: The lived experience of long COVID: A thematic analysis of an in-depth interview study
Source: PLOS Ment Health. 2026 Feb 6;3(2):e0000500. doi: 10.1371/journal.pmen.0000500 (PMC12880701; doi:10.1371/journal.pmen.0000500)
Supplement: S17 Table — (DOCX) [file pmen.0000500.s017.docx]

**S17 Table. Plans and Goals Codes**

| **Code:** | **Code Endorsement Range:** | **Code Description:** | **Example Quotes:** |
| --- | --- | --- | --- |
| **Plan/Goals** |  |  |  |
| **Personal** |  |  |  |
| No personal goals | 3 (8.8%) | Reported no personal goals at time of interview | “I don't really have many personal goals.” |
| Unchanged | 11 (32.4%) - 15 (44.1%) | Reported personal goals have remained unchanged since development of LC | “… my plans and goals have not changed.” |
| **Negatively changed** |  |  |  |
| Inability to continue living in same place | 4 (11.8%) | Reported inability to continue living in the same place since development of LC | “So I ended up coming down here so my parents could help because I was like running myself into the ground.” |
| Slower/adjusted progress | 19 (55.9%) - 20 (58.8%) | Reported slow/adjusted progress to personal goals and plans since development of LC | “I think maybe it just might be a little bit (more time) than maybe I had originally envisioned.” |
| Preparing for health decline | 3 (8.8%) | Reported preparation for health decline in regard to current goals/plans since development of LC | “I literally was… getting rid of my material things… trying to clean house because I really thought I was going to pass on.” |
| Activity levels | 9 (26.5%) - 10 (29.4%) | Reported decreased activity levels in regard to current goals/plans since development of LC | “We pared them back…we did a short trip that we did some hiking and we had to watch where we were hiking at with me.” |
| Ability to travel | 9 (26.5%) - 10 (29.4%) | Reported decreased ability to travel in regard to current goals/plans since development of LC | “And I want to be able to do that without being fatigued and being able to walk as you do when you're a tourist in different places.” |
| Friendships/Social | 4 (11.8%) - 7 (20.6%) | Reported negative changes/decreased ability to engage in friendships and social activities since development of LC | “I can't really make any plans with any friends I have.” |
| Romantic relationships | 3 (8.8%) - 5 (14.7%) | Reported negative changes/decreased ability to engage in romantic relationships since development of LC | “I would love to be dating, but I can’t really.” |
| Changed family plans | 2 (5.9%) - 6 (17.6%) | Reported negative changes/decreased ability to engage in family plans since development of LC | “I don't spend more than four hours watching my grandchildren by myself cause I get tired easily.” |
| **Positively changed** |  |  |  |
| Increased focus on health | 12 (35.3%) | Reported increased focus on personal health since development of LC | “I have to be a lot more careful about how I take care of my health because from now on, even if I get the flu, it's going to hit me harder than it would have before.” |
| Increased advocacy, research | 4 (11.8%) - 6 (17.6%) | Reported increased focus on/engagement in research or advocacy since development of LC | “Other than that, I guess I'm going to keep fighting and I've been joining the activist groups especially to attempt to get masking back in health care.” |
| Spend more time with family | 4 (11.8%) | Reported increased time spent with family since development of LC | “And I plan a family reunion for us because I'm in bed a lot, but like my mind works fairly well now.” |
| New projects/hobbies | 7 (20.6%) - 8 (23.5%) | Reported engagement in new projects/hobbies/activities since development of LC | “My next hobby I picked up is genealogy.” |
| More content | 1 (2.9%) - 4 (11.8%) | Reported feeling more content since development of LC | “… just figuring out how to be content with what I do have and what I can offer this lifetime.” |
| Finding Purpose | 2 (5.9%) | Reported finding purpose since development of LC | “I'm looking for a purpose of what can I do? I never got married and never had children and that was always what I thought I was going to be.” |
| **Career** |  |  |  |
| Changed career type | 3 (8.8%) - 5 (14.7%) | Reported changing career focus/type since development of LC | “And with future career-wise, I never thought I'd be in graphic design whatsoever.” |
| Unchanged | 7 (20.6%) - 8 (23.5%) | Reported no change in plans/goals due to developing LC | “Well, I'm going to plan to keep on working and just, um, to deal with this and work around it, you know, as much as I can...” |
| **Negatively changed** |  |  |  |
| Delay retirement | 1 (2.9%) | Reported delaying retirement due to developing LC |  |
| Quicken retirement | 2 (5.9%) - 5 (14.7%) | Reported quickening retirement due to developing LC | “And although we weren't financially ready to have this happen now, we'll have to, in the next few years, make the changes we need to, like if we need to sell our health, get something smaller, and whatever we need to do to modify for what our future may look like.” |
| Positively changed | 1 (2.9%) - 2 (5.9%) | Reported positive changes to personal goals/plans since developing LC | “I'm going to kind of stay where I'm at for a little while just to keep an income.” |
| Financial difficulties | 5 (14.7%) | Reported financial difficulties/concerns due to developing LC | “I haven't had income.” |
| Difficult making plans due to LC | 14 (41.2%) - 17 (50.0%) | Reported difficulty making plans/setting goals since developing LC | “… it's very hard to have goals...” |
