# Supplementary material for: The lived experience of long COVID: A thematic analysis of an in-depth interview study
Source: PLOS Ment Health. 2026 Feb 6;3(2):e0000500. doi: 10.1371/journal.pmen.0000500 (PMC12880701; doi:10.1371/journal.pmen.0000500)
Supplement: S18 Table — (DOCX) [file pmen.0000500.s018.docx]

**S18 Table. Prior Knowledge of Long COVID Codes**

| **Code:** | **Code Endorsement Range:** | **Code Description:** | **Example Quotes:** |
| --- | --- | --- | --- |
| **Prior Knowledge of LC** |  |  |  |
| Unsure | 1 (2.9%) | Uncertainty about knowledge of LC prior to developing LC | (Had you heard of long COVID before you began experiencing long-term symptoms?)  “I'm not sure, actually. I mean, I've heard, I've known about it for a while now, but whether, which came first, I'm not sure.” |
| **No** |  |  |  |
| Did Not Understand It | 0 (0.0%) - 2 (5.9%) | Did not understand LC or aspects of LC prior to developing LC | “Definitely sounded bad, but I didn't understand it at all. The extent of what can happen.” |
| Had Not Heard of It | 11 (32.4%) - 12 (35.3%) | Did not hear about LC prior to developing LC | “So we didn't really know long COVID was a thing. We just thought people were still sick.” |
| **Yes** |  |  |  |
| News/Media | 4 (11.8%) - 6 (17.6%) | Had engaged with news/media about LC prior to developing LC | “I got sick in November of 2020, and I think that there were just beginning to be news stories about people who weren't getting well.” |
| Just Heard of It | 15 (44.1%) - 16 (47.1%) | Had heard about LC in some capacity prior to developing LC | “Yes, I'd heard of long COVID…” |
| **Reports Incorrect Perception** |  |  |  |
| Symptom Type | 2 (5.9%) - 3 (8.8%) | Had heard of LC, but had an incorrect perception of the symptoms associated with LC prior to developing LC | “I guess I thought it was just people that never get better. And, and so they, they kind of continue to have the same symptoms as you get during COVID. So I think I probably thought it was more like a respiratory thing.” |
| Severity | 8 (23.5%) - 11 (32.4%) | Had heard of LC, but had an incorrect perception of the severity symptoms associated with LC prior to developing LC | “I perceived it to be people that had the more serious forms of COVID than what I had.” |
| Duration | 0 (0.0%) - 3 (8.8%) | Had heard of LC, but had an incorrect perception of the duration of LC prior to developing LC | “And I thought that it was temporary conditions that may only last a couple of months or longer depending on the person.” |
| Other | 2 (5.9%) - 5 (14.7%) | Had heard of LC, but had a generally incorrect perception of LC prior to developing LC | “I guess I thought it was probably just a continuation of the illness, which has been somewhat different than what my experience has been, at least.” |
| Personal Connection | 2 (5.9%) - 3 (8.8%) | Had heard of LC through a personal channel/knew someone struggling with LC prior to developing LC | “I have a friend… whose daughter was experiencing long COVID symptoms and so that was the only personal connection I've had.” |
| Researched/Read A Lot About It Prior | 1 (2.9%) | Had heard of LC and research or read a lot about LC prior to developing LC | “I've read about the long-term effects of COVID, certainly.” |
