# Supplementary material for: The lived experience of long COVID: A thematic analysis of an in-depth interview study
Source: PLOS Ment Health. 2026 Feb 6;3(2):e0000500. doi: 10.1371/journal.pmen.0000500 (PMC12880701; doi:10.1371/journal.pmen.0000500)
Supplement: S19 Table — (DOCX) [file pmen.0000500.s019.docx]

**S19 Table. Prior to Long COVID Codes**

| **Code:** | **Code Endorsement Range:** | **Code Description:** | **Example Quotes:** |
| --- | --- | --- | --- |
| **Prior to Long COVID (LC)** |  |  |  |
| Hobbies | 20 (58.8%) - 25 (73.5%) | Engagement in any hobbies/activities outside of social group, family, career, community activities prior to developing LC | “I really liked yard work… and I watched a lot of TV, movies, I read a lot… I am so far removed from all of those things now.” |
| Social Group Involvement | 4 (11.8%) - 8 (23.5%) | Engagement in any activities involving a social group/social interaction prior to developing LC | “At least once a year, I was out of the country, and then I also visited friends.” |
| School Involvement | 3 (8.8%) | Engagement in any activities involving school/academics prior to developing LC | “I went to school. That was a pretty big part of my time.” |
| Family Involvement | 13 (38.2%) - 14 (41.2%) | Engagement in any activities involving family/familial interaction prior to developing LC | “A major component of my life has been helping with taking care of my grandchildren.” |
| Career Involvement | 26 (76.5%) - 29 (85.3%) | Engagement in any career/job/school commitments or activities prior to developing LC | “Like I said, I was a nurse. I was working anywhere between, like, 40 to 60 to 80 hours.” |
| Community Involvement | 8 (23.5%) | Engagement in any community/volunteer/spiritual/religious activities prior to developing LC | “(I) got into some volunteer work before I got sick… with an animal rescue organization where my family had adopted several animals.” |
| Athlete/Active Lifestyle | 21 (61.8%) - 25 (73.5%) | Engagement in any athletics/frequent exercise or any activities that require physically active involvement prior to developing LC | “I think I participated in about eight half marathons before I got sick.” |
