# Supplementary material for: The lived experience of long COVID: A thematic analysis of an in-depth interview study
Source: PLOS Ment Health. 2026 Feb 6;3(2):e0000500. doi: 10.1371/journal.pmen.0000500 (PMC12880701; doi:10.1371/journal.pmen.0000500)
Supplement: S20 Table — (DOCX) [file pmen.0000500.s020.docx]

**S20 Table. Religious/Spiritual Ideology Codes**

| **Code:** | **Code Endorsement Range:** | **Code Description:** | **Example Quotes:** |
| --- | --- | --- | --- |
| **Religious/Spiritual Ideology** |  |  |  |
| **Unchanged** |  |  |  |
| Not religious (previously or currently) | 1 (2.9%) - 2 (5.9%) | Reported no change in religious ideology since developing LC due to not being religious prior to LC or currently | “I've not been a highly religious person, I guess.” |
| Not yet turned to religion | 0 (0.0%) - 1 (2.9%) | Reported no change in religious ideology since developing LC due to not utilizing religion as a support | “I'm going to say not yet. I think I've been in a state of... I'm just barely getting to the point of trying to think of it in the big picture sense as opposed to just reacting.” |
| Continued utilizing religion as support | 12 (35.3%) - 13 (38.2%) | Reported no change in religious ideology since developing LC due to continuing to utilize religion as a support both prior to LC and currently | “No, I'm a spiritual person but not a religious person and I don't go to church.” |
| Currently evolving | 2 (5.9%) | Reported no change in religious ideology since developing LC due to role of religion currently evolving within their life | “I very much lost all my faith. And it's something that I'm still trying to find again.” |
| **Decreased** |  |  |  |
| Resentment | 2 (5.9%) - 3 (8.8%) | Reported decreased religiosity due to religious resentment felt in developing LC | “Not much. I was, I wasn't really, really religious in the first place, so whenever I got sick, it was kind of like a resentment, so I just, I never really kind of went back.” |
| Increased | 2 (5.9%) - 3 (8.8%) | Reported increased religiosity since developing LC | “I'm probably about the same. I mean, it did get stronger in the beginning because I thought, you know, all these people are telling me just pray, pray, pray…” |
| **Perceived role of religion** |  |  |  |
| Something to believe in | 2 (5.9%) - 5 (14.7%) | Reported utilizing religion as a belief structure while managing LC | “Just having the belief that there's somebody out there looking out for me that I can talk to when I can't talk to anybody else just helps.” |
| Better attitude | 2 (5.9%) | Reported utilizing religion to improve mood/attitude while managing LC | (How do you think that would change if you weren't religious?)  “I'd probably have a much worse attitude.” |
| Reduced anger | 1 (2.9%) - 2 (5.9%) | Reported utilizing religion to reduce anger while managing LC | “I mean, I could be very, very, very angry about all this, and there are people that really are.” |
| Increased focus | 1 (2.9%) | Reported utilizing religion to increase focus while managing LC | “Yeah, it's kind of strengthened it in the sense that I'm really focusing on that… I just am deeper into it now and feeling it, understanding it, and using those tools as well.” |
| Coping strategy | 5 (14.7%) | Reported utilizing religion as a coping strategy while managing LC | “I have several very good friends that are pastors and just, you know, been able to reach out to them and have the support of my church family and things when I've had sicknesses or when I've had COVID and things.” |
| Gives perspective | 3 (8.8%) - 4 (11.8%) | Reported utilizing religion to gain perspective while managing LC | “And really in the end, you're on this earth for such a short time, there's only a few things that matter and it's probably not your health. Some things you do here on earth, there are things you gain that doesn't matter, really.” |
| Reason to keep living | 1 (2.9%) - 2 (5.9%) | Reported utilizing religion as a reason to keep living/fighting while managing LC | “(If I were not religious) I'd probably not give a hoot if I die or not. I think it helps to be religious.” |
