# Supplementary material for: The lived experience of long COVID: A thematic analysis of an in-depth interview study
Source: PLOS Ment Health. 2026 Feb 6;3(2):e0000500. doi: 10.1371/journal.pmen.0000500 (PMC12880701; doi:10.1371/journal.pmen.0000500)
Supplement: S21 Table — (DOCX) [file pmen.0000500.s021.docx]

**S21 Table. Symptom Fluctuation Codes**

| **Code:** | **Code Endorsement Range:** | **Code Description:** | **Example Quotes:** |
| --- | --- | --- | --- |
| **Sx fluctuation** |  |  |  |
| **Predict good/bad symptom day** |  |  |  |
| Can predict | 1 (2.9%) | Reported ability to predict good/bad LC symptom days | “I haven't had a really bad migraine in a while, but I think I've also just gotten way more attuned to knowing kind of what the onset are and I can catch them early enough.” |
| Cannot predict | 6 (17.6%) - 10 (29.4%) | Reported inability to predict good/bad LC symptom days | “And there's just no predicting when it's not going to be a good day anymore for me.” |
| **Sx triggers/relief** |  |  |  |
| Unable to identify triggers/relieving factors | 12 (35.3%) | Reported inability to identify triggers/relieving factors regarding LC symptoms | “This week they seem to be worse, but other weeks I'm good. Yeah, (I) just can't really tie it to anything.” |
| **Worsen** |  |  |  |
| Time of year | 2 (5.9%) | Reported time of year to worsen LC symptoms | (Do you feel like you do feel worse in the winters with long COVID symptoms as well?)  “Yeah, when it's dark, I just want to sleep.” |
| Emotional exertion | 3 (8.8%) - 4 (11.8%) | Reported emotional exertion to worsen LC symptoms | “And since then, it always happens when I use a certain amount of energy that I have not yet figured out. But I know that it also happens with mental exertion and emotional exertion.” |
| Mental exertion | 10 (29.4%) - 12 (35.3%) | Reported mental exertion to worsen LC symptoms | “But I find that I really put a limit on how much mental stuff I do.” |
| Heat | 1 (2.9%) - 4 (11.8%) | Reported heat to worsen LC symptoms | “… if I'm out in the heat, but that might, you know, exacerbate things.” |
| Social interaction | 3 (8.8%) - 4 (11.8%) | Reported social interaction to worsen LC symptoms | “And if I'm socializing, I've got an hour or two. The more people, the less time I have.” |
| Food-related | 4 (11.8%) - 7 (20.6%) | Reported food/food-related impacts to worsen LC symptoms | “I really don't eat that much anymore either because it seems like a lot of a lot of food triggers different symptoms.” |
| Physical exertion/exercise | 16 (47.1%) | Reported physical exertion/exercise to worsen LC symptoms | “I've got what they call exercise intolerance.” |
| Poor sleep | 1 (2.9%) - 3 (8.8%) | Reported poor sleep to worsen LC symptoms | “If I get a good night's sleep, it's better the next day...” |
| Allergies | 1 (2.9%) | Reported allergies to worsen LC symptoms | “And so that affects other conditions when my allergies are worse.” |
| Life stress | 2 (5.9%) - 3 (8.8%) | Reported life stress to worsen LC symptoms | “It's pretty present all the time, but it does get so much worse if there's any sort of stress at all” |
| **Improve** |  |  |  |
| Laying Down | 5 (14.7%) - 8 (23.5%) | Reported laying down to improve LC symptoms | “The fatigue is constant. I can't really do much of anything for more than like an hour before I have to like lay down in the dark, close my eyes, you know, get rid of all the stimulus.” |
| Other | 0 (0.0%) - 3 (8.8%) | Reported action/environmental factor/activity/etc. to improve LC symptoms | “Just being in the energy of nature I think helps.” |
| **Fluctuating** |  |  |  |
| Some sx better, others worse | 6 (17.6%) - 9 (26.5%) | Reported fluctuation in LC symptoms, in which some symptoms have improved and others have not | “Like, the chest pain is still here… but it's not as bad, but I still have other symptoms that are way worse.” |
| Severity/Frequency Changes | 29 (85.3%) - 30 (88.2%) | Reported fluctuation in LC symptoms, in which symptoms fluctuated inn severity and/or frequency or the pattern of fluctuation was unclear | “So anyway, I do feel better (with) the pain than other days where I don't feel well at all.” |
| **Static** |  |  |  |
| Remains steady throughout the day | 2 (5.9%) - 3 (8.8%) | Reported stability of LC symptoms throughout the day | “Depression is here 24-7. It doesn't matter how I'm feeling. I feel depressed.” |
| Remains steady day to day | 11 (32.4%) - 14 (41.2%) | Reported stability of LC symptoms day to day | “… they don't really come and go, they're pretty static.” |
