# Supplementary material for: The lived experience of long COVID: A thematic analysis of an in-depth interview study
Source: PLOS Ment Health. 2026 Feb 6;3(2):e0000500. doi: 10.1371/journal.pmen.0000500 (PMC12880701; doi:10.1371/journal.pmen.0000500)
Supplement: S22 Table — (DOCX) [file pmen.0000500.s022.docx]

**S22 Table. Treatments for Long COVID Codes**

| **Code:** | **Code Endorsement Range:** | **Code Description:** | **Example Quotes:** |
| --- | --- | --- | --- |
| **Treatments for LC** |  |  |  |
| Technology (watches, etc.) | 3 (8.8%) - 5 (14.7%) | Reported utilizing technology (smart watches, etc.) for treatment of LC symptoms | “My physical therapist had me get a Fitbit.” |
| Medication/Supplement | 19 (55.9%) | Reported utilizing medication and/or supplements for treatment of LC symptoms | “And so I now have to take beta blockers to keep my heart from racing.” |
| Physical Therapy | 3 (8.8%) - 5 (14.7%) | Reported engaging in physical therapy for treatment of LC symptoms | “When I left the hospital, I also had appointments for physical therapy.” |
| Hyperbaric oxygen (HBOT) | 1 (2.9%) | Reported utilizing hyperbaric oxygen (HBOT) for treatment of LC symptoms | “Other people try hypobaric oxygen therapy, HBOT.” |
| Medical spas | 0 (0.0%) - 1 (2.9%) | Reported attending medical spas for treatment of LC symptoms | “You have to go to one of the medical spas and pay a ton of money that's overpriced but it works.” |
| Saline/IV | 3 (8.8%) - 4 (11.8%) | Reported utilizing saline and/or IV infusions for treatment of LC symptoms | “Some important things like IV vitamin and saline infusion. That helps me a lot.” |
| CBD | 2 (5.9%) | Reported utilizing CBD/THC for treatment of LC symptoms | “I've tried… CBD.” |
| Exercise | 1 (2.9%) - 3 (8.8%) | Reported engaging in exercise for treatment of LC symptoms | “Intervals. So I would go on the bike for 30 seconds and get the heart rate up a little bit more and then I would stop, I would sit in a chair and kind of meditate to get my heart rate back down and I would repeat that a couple of times and it was kind of just training my system to go up and down.” |
| Diets | 5 (14.7%) - 6 (17.6%) | Reported utilizing dieting/diets for treatment of LC symptoms | “I started intermittent fasting.” |
| Hospitalization | 1 (2.9%) - 7 (20.6%) | Reported hospitalization for treatment and management of LC symptoms | “But I have been in the hospital several times now to treat it.” |
| Travel internationally for treatment | 2 (5.9%) | Reported travelling internationally for treatment of LC symptoms | “For treatment, I had some great treatment in (Europe), which really helped and I have paid an arm and a leg for very specialized doctors who deal with (long COVID) and I've never really had an issue.” |
| Compression | 0 (0.0%) - 3 (8.8%) | Reported utilizing compression techniques for treatment of LC symptoms | “I have to do compression and electrolytes and things for that.” |
| Acupressure/Acupuncture | 2 (5.9%) - 3 (8.8%) | Reported utilizing acupressure and/or acupuncture for treatment of LC symptoms | “It's more of an activity and I would call it medical… acupuncture or acupressure.” |
| Inhaler | 0 (0.0%) - 2 (5.9%) | Reported utilizing an inhaler for treatment of LC symptoms | “I was still really having issues with shortness of breath, so I went into (clinic) and they were like, yeah, no, it's probably just still because of the COVID, here's an inhaler to help with the shortness of breath.” |
| Allergy shots | 1 (2.9%) | Reported receiving allergy shots for treatment of LC symptoms | “And the allergist tests me on allergy shots.” |
| Occupational therapy | 1 (2.9%) - 2 (5.9%) | Reported engaging in occupational therapy for treatment of LC symptoms | “I also had appointments for physical therapy, occupational therapy...” |
| Speech therapy | 1 (2.9%) - 3 (8.8%) | Reported engaging in speech therapy for treatment of LC symptoms | “The neurologist sent me for vestibular therapy and for cognitive speech therapy.” |
| Ozone | 1 (2.9%) | Reported utilizing ozone for treatment of LC symptoms | “I might do this ozone treatment and I'm trying to decide where I go for it somewhere overseas because it's much cheaper.” |
| Botox | 1 (2.9%) | Reported receiving Botox for treatment of LC symptoms | “… they tried Botox.” |
| Lidocaine injections | 1 (2.9%) | Reported receiving lidocaine injections for treatment of LC symptoms | “They did some like lidocaine injections around my neck and into like the back of my head to see if that helped.” |
| Other | 7 (20.6%) - 8 (23.5%) | Reported utilizing another treatment for treatment of LC symptoms | “It's called a CO2 bath.” |
