# Supplementary material for: The lived experience of long COVID: A thematic analysis of an in-depth interview study
Source: PLOS Ment Health. 2026 Feb 6;3(2):e0000500. doi: 10.1371/journal.pmen.0000500 (PMC12880701; doi:10.1371/journal.pmen.0000500)
Supplement: Attachment — Submitted filename: LCqual_response6_reviewer.docx [file pmen.0000500.s024.docx]

David Onchonga, PhD

Academic Editor

*PLOS Mental Health*

Dear Dr. Onchonga,

We are submitting our revised manuscript “The lived experience of long COVID: A thematic analysis of an in-depth interview study” for reconsideration for publication in *PLOS Mental Health.* We have responded to each reviewer comment below. The authors thank the associate editor and reviewers for the careful review and thoughtful comments.

Journal Requirements:

1. Please send a completed 'Competing Interests' statement, including any COIs declared by your co-authors. If you have no competing interests to declare, please state "The authors have declared that no competing interests exist". Otherwise please declare all competing interests beginning with the statement "I have read the journal's policy and the authors of this manuscript have the following competing interests:"

*The sentence “The authors have declared that no competing interests exist” has been added to the “Competing Interests” section of the submission portal.*

2. Please amend your detailed Financial Disclosure statement. This is published with the article. It must therefore be completed in full sentences and contain the exact wording you wish to be published.

If you did not receive any funding for this study, please simply state: “The authors received no specific funding for this work.”

*The sentence “The authors received no specific funding for this work” has been added to the “Financial Disclosure” section of the submission portal.*

3. Please insert an Ethics Statement at the beginning of your Methods section, under a subheading 'Ethics Statement'. It must include:

1) The name(s) of the Institutional Review Board(s) or Ethics Committee(s)

2) The approval number(s), or a statement that approval was granted by the named board(s)

3) (for human participants/donors) - A statement that formal consent was obtained (must state whether verbal/written) OR the reason consent was not obtained (e.g. anonymity). NOTE: If child participants, the statement must declare that formal consent was obtained from the parent/guardian.

*We have added a subheading “Ethics Statement” including this information:*

*“Ethics Statement*

*The project was approved by the Iowa State University Institutional Review Board (IRB ID: 23-158). Formal written or verbal informed consent was not required by the Iowa State University Institutional Review Board (IRB) as this project was deemed exempt. Participants read through a consent information sheet and indicated consent by clicking forward on the screen. Participants were encouraged to reach out to the research team with questions or concerns at any point during the research process. Participants also gave verbal consent for recording of their interview prior to beginning the interview. The survey was completed prior to the interview in all cases to allow participants to read information about the study and decide whether they would like to participate prior to engagement in the interview process.”* (page 5, lines 95-105)

4. Please provide separate figure files in .tif or .eps format.

*The Figure 1 file is now attached in .tiff format.*

5. We have noticed that you have uploaded Supporting Information files, but you have not included a list of legends. Please add a full list of legends for your Supporting Information files after the references list.

*We have provided a full list of legends following the references section.* (page 33, lines 743-765)

6. If the reviewer comments include a recommendation to cite specific previously published works, please review and evaluate these publications to determine whether they are relevant and should be cited. There is no requirement to cite these works unless the editor has indicated otherwise.

*We have followed these instructions.*

Reviewer #1: I greatly appreciate the topic as well as your choice of a qualitative approach, which constitutes an effective methodology for gaining a deeper understanding of the subjective experiences of long COVID patients.

However, there may be concerns regarding significant biases related to the sample. The experiences of individuals who are able to communicate and who have access to telephones and the internet are likely to differ substantially from those of individuals who are unable to communicate or who lack the necessary access to these technologies. Other characteristics of the sample—particularly gender and ethnicity—might also indicate a systematic bias. An important question is why participant self-selection occurred in this manner and how the sample differs from the broader population of patients with long COVID.

*Thank you for this comment. We have expanded on the limitations noted in the discussion:*

*“In this study, participants opted into the interview process voluntarily. This may introduce self-selection bias, such that individuals with more severe long COVID experiences or stronger opinions regarding long COVID may have been more likely to participate. However, individuals with very severe symptoms may not have been able to participate in the study and may not have their experiences represented. Participants were not asked about their motivation to participate in the interviews, though this motivation may influence participants’ likelihood of taking part in the study. Participants were recruited from a prior survey study that involved recruitment through social media and mass email, and individuals without access to these resources may not have been included. In addition, individuals with more social engagement with their illness experience may be particularly likely to be recruited from social media, biasing the participating sample. We utilized snowball sampling, and it is possible that participants may be more homogenous than the larger long COVID population given preexisting similarities between participants. Additionally, we did not require that participants provide medical records to verify diagnosis, which may have introduced inconsistency in the reported findings. It is possible that individuals reporting long COVID may be experiencing symptoms due to another medical condition or process. However, there is not an established diagnostic criteria for long COVID and reports of long COVID often depend on patient self-report of symptoms [41].”* (page 27, lines 544-560)

*“The homogenous participant demographic characteristics further limits the generalizability of the findings. To participate in the study, participants had to have access to the internet and a telephone, and individuals without access to these resources would have been excluded from the study, limiting generalizability. (page 27, lines 562-566)*

*Our participants predominantly identified as female, white and not Hispanic or Latino/a/e, limiting interpretation to populations beyond this sample. Other work centered on long COVID has argued for the importance of promoting diversity and inclusion of diverse sociodemographic characteristics and experiences, with success incorporating community- and patient-engaged models [39,42]. Further work investigating the lived experience of long COVID from the experience of a diverse set of individuals with long COVID may provide additional insight into the variability of the impacts of long COVID. Sociodemographic characteristics such as race and ethnicity have been associated with stigma and health disparities [43], indicating the importance of considering how these factors influence the long COVID illness experience. Non-Hispanic Black and Hispanic adults were noted to have significantly higher odds of medical mistrust compared to non-Hispanic White individuals in a survey completed prior to the COVID-19 pandemic [44], emphasizing that sociodemographic characteristics and lived experience related to other identities must be considered when evaluating the experiences of individuals with long COVID. It will be important for future research to include a demographically diverse and representative sample in order to provide comprehensive insight on the needs within long COVID support services.”* (pages 28, lines 567-582)

The severity of symptoms may also be an issue. It is possible that individuals with more severe long COVID symptoms or stronger opinions about the condition may have been more likely to participate. Conversely, individuals with very severe symptoms may not have had the capacity to take part in any research.

*Thank you for this note. We have expanded on this in the limitations section of the discussion:*

*“In this study, participants opted into the interview process voluntarily. This may introduce self-selection bias, such that individuals with more severe long COVID experiences or stronger opinions regarding long COVID may have been more likely to participate. However, individuals with very severe symptoms may not have been able to participate in the study and may not have their experiences represented.”* (page 27, lines 544-548)

On the other hand, I do not consider the small sample size or the limited generalizability of the findings to be problematic, given that this is a qualitative study.

*Thank you.*

Considering the exploratory nature of the study, I would have expected the interviews to be less structured. Additionally, the use of percentages in the text is somewhat confusing.

*Thank you for these thoughts. We have removed percentages from the Results text to avoid confusion. As we anticipated recruiting all participants that were interested in interviews from our initial study, we focused on standardizing our interview guide to allow collection of similar data across participants. However, the use of follow up questions allowed more exploration related to the lived experience of individuals interviewed.*

More importantly, long COVID is an umbrella term encompassing various symptoms. The lived experience of each individual is inevitably shaped by their specific symptoms or combinations of symptoms. A stronger focus on the impact of particular symptoms on patients’ lives could provide a much clearer understanding of their experiences.

*Thank you for this comment. Participants in our study were not asked about the impact of specific long COVID symptoms and their impact on the lived experience, as we instead asked broadly about the experience of long COVID. While some participants chose to share more detailed information about the impact of specific long COVID symptoms, not all participants shared this information. As we wish to avoid prioritizing the lived experience of some participants over others, we have chosen not to investigate specific symptoms at the individual level. As a thematic analysis of the group of participants as a whole, we have instead focused on larger themes incorporating common themes of lived experience among individuals with long COVID. Future investigations could explore the impact of specific symptoms on long COVID lived experience in more detail.*

Good luck with your further research!

*Thank you for your thoughtful review.*

Reviewer #2: Introduction

1. While the introduction establishes the need for research into lived experience, it does not adequately review prior qualitative studies (if any) on long COVID or comparable post-viral conditions (e.g., ME/CFS). Positioning this study within a qualitative research landscape would enhance its originality claim.

*Thank you for your comment. We have expanded on the relevant qualitative literature in the introduction, to better position our paper in the long COVID qualitative literature landscape:*

“*There are nuanced biopsychosocial impacts of long COVID, including living with chronic physical and mental health concerns, slow rehabilitation and recovery, limited knowledge of long-term effects of Sars-CoV-2 infection, limited social support, limited access to healthcare services, and unsatisfactory experiences with healthcare providers [10]. These biopsychosocial impacts have been outlined in prior meta-analyses of qualitative study of long COVID experience, with the need for increased representation of North American samples [10]. Other literature shows the heterogeneous nature of the long COVID experience, and highlights the continued need for qualitative research that gives voice to the specific experiences of those living with long COVID illness. Furthermore, prior research has emphasized the necessity of connecting individuals with long COVID to various resources and systems of support, based on participant identified barriers to healing [11,12]. These experiences and long-term impacts, alongside physical and mental health symptoms, as well as the continued need for supportive resources, poses a need for further understanding of individual experiences of long COVID to more effectively treat and support those impacted by long COVID. Such investigations will provide essential information about the mental, social, and physical health impacts of long COVID, and this information is necessary to develop and test interventions.* (pages 4, lines 64-79)

2. The prevalence statistics provided are quite broad and could be problematized—what accounts for this variation (different populations, definitions of long COVID, time frames)? A brief clarification would improve scientific rigor.

*Thank you for your comment. We have expanded on the variance between the prevalence*	*statistics provided in the introduction:*

*“It has been estimated that between 6% and 39% of individuals experience prolonged symptoms after acute Sars-CoV-2 infection [4,5]. Suggested prevalence rates of long COVID have varied substantially, influenced by the novelty and multisystem impacts of the condition [4,5]. Past literature has varied regarding the criteria used to define long COVID, further contributing to the variation in prevalence noted. [4,5].* (page 3, lines 52-56)

3. The introduction states the aim to study "lived experiences," but does not define what is meant by this term or how it is conceptually grounded (e.g., phenomenology, medical sociology, illness narratives). Clarifying this would strengthen the theoretical foundation.

*Thank you for this comment. We have expanded on the conceptual grounding in our decision to study “lived experiences” in our introduction:*

*“The aim of this qualitative study was to examine the lived experiences of those with self-reported long COVID, as prior research has shown the benefit of taking a phenomenological approach in understanding chronic illness [13]. Considering the lived experience, related to the subjective experience of an individual including psychological factors such as emotions, goals, and perceptions of an individual at a given point in their lives, had particular advantages for our study [14]. In alignment with phenomenological research, our study hopes to gather information on patterns of experiences among people with self-reported long COVID, through highlighting individual experiences [15].”* (page 5, lines 80-87)

4. The introduction leans heavily on the biopsychosocial framing but does not make explicit why this model is chosen over others (e.g., disability studies frameworks, social determinants of health perspectives). Making this explicit could broaden interpretive richness.

*Thank you for this comment. We have expanded on the choice to utilize a biopsychosocial framework in the introduction:*

“*In addition, the study seeks to explore the biopsychosocial impacts of long COVID, gaining a deeper understanding of the functional, quality of life, and existential impacts of long COVID from the perspective of those directly impacted. This biopsychosocial framework was adopted in addition to the phenomenological approach, to highlight the systemic and compounding impacts of biological, social and psychological experiences of those with self-reported long COVID [16]. The combination of these two approaches allows for a more holistic view of the long COVID experience.”* (page #5, lines #87-93)

Methods

1. The reliance on self-reported long COVID may reduce clinical validity, as no medical confirmation was sought.

2. This limitation is common in qualitative health studies but should be explicitly problematized.

*Thank you for this comment. We agree that depending on self-report of long COVID has limitations but also note that there is no diagnostic test for long COVID complicating all efforts to precisely define and diagnose the condition. We have added a note regarding the limitations of self-reporting long COVID to our Discussion section:*

*“Additionally, we did not require that participants provide medical records to verify diagnosis, which may have introduced inconsistency in the reported findings. It is possible that individuals reporting long COVID may be experiencing symptoms due to another medical condition or process. However, there is not an established diagnostic criteria for long COVID and reports of long COVID often depend on patient self-report of symptoms [41].”* (page 27, lines 554-559)

3. Recruiting from social media and a prior survey may overrepresent individuals with more severe or socially engaged illness experiences.

*We have added to our Discussion that recruitment from social media and mass email in the prior survey our participants were recruited from may impact the representation of our sample:*

*“Participants were recruited from a prior survey study that involved recruitment through social media and mass email, and individuals without access to these resources may not have been included. In addition, individuals with more social engagement with their illness experience may be particularly likely to be recruited from social media, biasing the participating sample.”* (page 27, lines 549-552)

4. Snowball sampling further risks homogeneity in participant perspectives.

*Thank you for noting this. We have added this limitation to the Discussion section. The Discussion now reads:*

*“We utilized snowball sampling, and it is possible that participants may be more homogenous than the larger long COVID population given preexisting similarities between participants.”* (page 27, lines 552-554)

5. The methods do not mention efforts to ensure diversity across demographics (e.g., race, socioeconomic status), which is important in understanding the varied impact of long COVID.

*Thank you for this comment and the authors agree that ensuring sociodemographic diversity is important to generalizability to the general long COVID population. The design of the study limited our possible sociodemographic diversity to those who participated in the prior survey. We have emphasized the importance of our limitation in our Discussion section as well as expanded on the possible differential experience of individuals with long COVID by sociodemographic characteristics.*

6. The authors note that code saturation was not reached, but themes did emerge.

7. While they cite literature suggesting saturation is not essential, a clearer justification of how they ensured analytical adequacy despite new codes emerging until the end would strengthen credibility.

*Thank you for this comment. We have updated the text to emphasize that there were subcodes added with each set of 5 interviews, we have emphasized that primary codes and final themes had emerged prior to the final set of interviews. This is relatively expected given the significant heterogeneity among individuals with long COVID. This section of the Methods section now reads:*

*“Although code saturation was assessed after every 5 interviews, saturation was not met, with each set of 5 interviews providing at least one new subcode, perhaps reflecting the heterogenous nature of long COVID [18–20]. However, it has been argued that saturation may not be required for impactful qualitative research [21]. Despite not reaching subcode-level saturation, all primary codes and final themes had emerged prior to the final set of interviews.”* (page 8, lines 137-142)

8. Although exempt status was obtained, relying solely on participants clicking forward on a screen could be seen as minimal consent.

*Thank you for this note. We have further emphasized that participants were encouraged to reach out to the research team with questions or concerns at any point in the research process. Our method of providing study information and obtaining informed consent was approved by the Institutional Review Board, additionally emphasized in our Ethics Statement:*

*“The project was approved by the Iowa State University Institutional Review Board (IRB ID: 23-158). Formal written or verbal informed consent was not required by the Iowa State University Institutional Review Board (IRB) as this project was deemed exempt. However, consent was acknowledged prior to completing the interview by clicking forward on an electronic screen detailing study information, as approved by the IRB. Participants were encouraged to reach out to the research team with questions or concerns at any point during the research process. Participants also gave verbal consent to recording of their interview prior to beginning the interview. The survey was completed prior to the interview in all cases to allow participants to read information about the study and decide whether they would like to participate prior to engagement in the interview process. Participants were free to skip any questions that they did not wish to answer during both the survey and the interview.”* (page 5-6, lines 96-105)

9. A stronger case could have been made for verbal consent at the beginning of interviews.

*We have now emphasized that all participants were provided with study information prior to completing the survey and the interview, with the opportunity to ask questions or address concerns with the research team. The survey was completed prior to the interview for all participants to allow participants to read through information regarding the study and make an informed decision about whether they wished to participate in the survey and interview. We have now emphasized this in our Ethics Statement:*

*“The project was approved by the Iowa State University Institutional Review Board (IRB ID: 23-158). Formal written or verbal informed consent was not required by the Iowa State University Institutional Review Board (IRB) as this project was deemed exempt. However, consent was acknowledged prior to completing the interview by clicking forward on an electronic screen detailing study information, as approved by the IRB. Participants were encouraged to reach out to the research team with questions or concerns at any point during the research process. Participants also gave verbal consent to recording of their interview prior to beginning the interview. The survey was completed prior to the interview in all cases to allow participants to read information about the study and decide whether they would like to participate prior to engagement in the interview process.”* (page 5-6, lines 96-105)

10. The methods do not describe what demographic data were collected (age, gender, race/ethnicity, socioeconomic background).

*Thank you for noting this omission. We have now added the specific sociodemographic characteristics collected through the accompanying survey, including age, gender, race, ethnicity, highest level of education, and income. This information is now presented in the Methods section:*

*“Inclusion criteria included being ≥18 years old, being from the United States, English verbal communication abilities, and reliable internet and telephone service and devices. Prior to the interview, participants responded to a survey assessing sociodemographic characteristics such as age, gender, race, ethnicity, highest level of education, and income as well as several physical and mental health characteristics.”* (page 7, lines 118-122)

11. Since the study explores lived experience, demographic and contextual diversity are highly relevant for interpreting findings.

*Our sample was relatively homogenous, with a relatively high proportion of white, non-Hispanic, and affluent individuals participating. Given the limited sociodemographic characteristics of our sample, it will be vital for further studies to investigate the lived experience of long COVID among a more diverse sample. We have noted this limitation as well as the importance of greater diversity in the future in our Discussion section:*

*“Our participants predominantly identified as female, white and not Hispanic or Latino/a/e, limiting interpretation to populations beyond this sample. Other work centered on long COVID has argued for the importance of promoting diversity and inclusion of diverse sociodemographic characteristics and experiences, with success incorporating community- and patient-engaged models [39,42]. Further work investigating the lived experience of long COVID from the experience of a diverse set of individuals with long COVID may provide additional insight into the variability of the impacts of long COVID. Sociodemographic characteristics such as race and ethnicity have been associated with stigma and health disparities [43], indicating the importance of considering how these factors influence the long COVID illness experience. Non-Hispanic Black and Hispanic adults were noted to have significantly higher odds of medical mistrust compared to non-Hispanic White individuals in a survey completed prior to the COVID-19 pandemic [44], emphasizing that sociodemographic characteristics and lived experience related to other identities must be considered when evaluating the experiences of individuals with long COVID. It will be important for future research to include a demographically diverse and representative sample in order to provide comprehensive insight on the needs within long COVID support services.”* (page 28, lines 566-581)

Results

1. The sample is overwhelmingly white (97.1%), highly educated (79.4% with bachelor’s or higher), and affluent (47.1% earning >$100k).

2. This homogeneity limits transferability of findings, as the lived experience of long COVID may differ significantly across racial, socioeconomic, and cultural contexts.

*Thank you for this note. We have noted the homogeneity of our sample in our limitations and have noted that future research should work to incorporate more heterogenous samples to better represent a fuller picture of the experience of individuals living with long COVID. Our Discussion section now includes:*

*“To participate in the study, participants had to have access to the internet and a telephone, and individuals without access to these resources would have been excluded from the study, limiting generalizability.*

*Our participants predominantly identified as female, white and not Hispanic or Latino/a/e, limiting interpretation to populations beyond this sample. Other work centered on long COVID has argued for the importance of promoting diversity and inclusion of diverse sociodemographic characteristics and experiences, with success incorporating community- and patient-engaged models [39,42]. Further work investigating the lived experience of long COVID from the experience of a diverse set of individuals with long COVID may provide additional insight into the variability of the impacts of long COVID. Sociodemographic characteristics such as race and ethnicity have been associated with stigma and health disparities [43], indicating the importance of considering how these factors influence the long COVID illness experience. Non-Hispanic Black and Hispanic adults were noted to have significantly higher odds of medical mistrust compared to non-Hispanic White individuals in a survey completed prior to the COVID-19 pandemic [44], emphasizing that sociodemographic characteristics and lived experience related to other identities must be considered when evaluating the experiences of individuals with long COVID. It will be important for future research to include a demographically diverse and representative sample in order to provide comprehensive insight on the needs within long COVID support services.”* (pages 27-28, lines 563-581)

3. The frequent use of percentages (e.g., “23.5–26.5%,” “61.8–79.4%,” etc.) risks reducing qualitative nuance into quasi-quantitative reporting.

4. While this conveys prevalence, it can distract from the depth of narrative insight.

5. A more interpretive synthesis—prioritizing meaning over frequency—would align more strongly with thematic analysis principles.

*Thank you for this comment. We have removed the percentages from the Results section to avoid confusion, instead emphasizing the narrative components of our work. We have maintained percentages within our Supplemental Material for readers who may be interested in these ranges.*

6. Some themes (e.g., Decreased Autonomy) are richly developed with layered subthemes, while others (e.g., Changes in Worldview) are less elaborated in the provided excerpt.

7. This imbalance risks privileging some experiences over others.

*Thank you for this comment. We acknowledge there are differences in length across the subthemes. This was a result of content differences, and inclusion decisions while reviewing the data. In the comparison of Decreased Autonomy to Changes in Worldview, we saw much more agreement and overlap in participant reports related to changes in worldview. Whereas, in discussing decreased autonomy, we saw more variance between participants due to the heterogeneous nature of symptom profiles and functional impacts. Additionally, for Changes in Worldview specifically, we attempted to be very intentional with subthemes included within the manuscript, as to avoid confounding factors such as political climate and pandemic experiences, that may have contributed to participant responses to worldview inquiries. We attempted to report only on changes to worldview that participants attribute to long COVID. As this manuscript is exploratory in nature, we hope further research will expand on these experiences.*

8. The results focus on symptoms, autonomy, and trust but do not address how gender, race, class, or disability identity shape lived experience.

9. Given the sample’s demographic skew, the absence of reflection on this limitation is notable.

*Thank you for this comment. We have expanded on the limitations of our sample’s sociodemographic limitations in our Discussion section, as well as indicated the importance of future work pursuing more demographically diverse and representative samples. The Discussion section now includes:*

*“To participate in the study, participants had to have access to the internet and a telephone, and individuals without access to these resources would have been excluded from the study, limiting generalizability.*

*Our participants predominantly identified as female, white and not Hispanic or Latino/a/e, limiting interpretation to populations beyond this sample. Other work centered on long COVID has argued for the importance of promoting diversity and inclusion of diverse sociodemographic characteristics and experiences, with success incorporating community- and patient-engaged models [39,42]. Further work investigating the lived experience of long COVID from the experience of a diverse set of individuals with long COVID may provide additional insight into the variability of the impacts of long COVID. Sociodemographic characteristics such as race and ethnicity have been associated with stigma and health disparities [43], indicating the importance of considering how these factors influence the long COVID illness experience. Non-Hispanic Black and Hispanic adults were noted to have significantly higher odds of medical mistrust compared to non-Hispanic White individuals in a survey completed prior to the COVID-19 pandemic [44], emphasizing that sociodemographic characteristics and lived experience related to other identities must be considered when evaluating the experiences of individuals with long COVID. It will be important for future research to include a demographically diverse and representative sample in order to provide comprehensive insight on the needs within long COVID support services.”* (pages 27-28, lines 563-581)

10. participants describe long COVID as a disability, yet the analysis does not explore the social or political implications of this identity shift.

11. This could have been developed more fully, perhaps linking to existing disability studies literature.

*Thank you for this comment. We have expanded on the impact of long COVID beyond physical symptoms, impacting identities, social relationships, and an individual’s ability to perform activities of daily living. This is now noted in our Discussion section:*

“*The substantial and disabling nature of long COVID symptoms was frequently noted throughout the interviews. In addition to the individual impacts of symptoms, participants often noted a change in their autonomy and function, sometimes noting that they now consider themselves to have a disability. Disability can be a social identity, indicating potential implications beyond the immediate impact of physical and mental health symptoms experienced related to long COVID. Other studies in the context of long COVID have emphasized the expansive nature of disability that can affect individuals, with the impact of physical symptoms compounded by barriers to social inclusion, cognitive symptoms, challenges in completing activities of daily living, as well as mental and emotional health problems [39]. Others with long COVID have noted the social implications of long COVID as an invisible disability, noting that others often are not accepting and understanding [40], influencing social relationships and the lived experience. Long COVID has been noted to substantially affect an individual’s sense of self, often with notable identity loss [40], indicating that long COVID can impact much beyond the physical symptoms. Although some individuals with long COVID may note disability, it is also important to note that others may not identify as an individual with a disability.”* (pages 25, lines 510-524)

Discussion

1. The discussion occasionally frames the study’s findings as broadly representative of “individuals with long COVID,” despite acknowledging severe demographic skew and sample limitations later.

*Thank you for this comment. We have adjusted language referring specifically to our sample to note that these findings refer specifically to the sample of individuals participating in our study. We have utilized the language “this sample of individuals with long COVID” to emphasize this limitation. Given the substantial variance of symptoms and severity noted among individuals with long COVID as well as the limited sociodemographic characteristics of our sample, we feel that this change in phrasing better represents our sample and results.*

2. While the authors note demographic homogeneity, they do not explore how gender, race, or socioeconomic background might shape experiences of mistrust, stigma, or adaptation.

3. Given the sample characteristics, this omission leaves an important analytical gap.

*Thank you for this note. We have expanded on this limitation in our Discussion section, noting the importance of considering sociodemographic characteristics in the context of medical mistrust and discrimination. While our sample had limited sociodemographic diversity, it will be important for future research to work to recruit a demographically diverse set of participants. This section of our Discussion section now reads:*

*“Other work centered on long COVID has argued for the importance of promoting diversity and inclusion of diverse sociodemographic characteristics and experiences, with success incorporating community- and patient-engaged models [39,42]. Further work investigating the lived experience of long COVID from the experience of a diverse set of individuals with long COVID may provide additional insight into the variability of the impacts of long COVID. Sociodemographic characteristics such as race and ethnicity have been associated with stigma and health disparities [43], indicating the importance of considering how these factors influence the long COVID illness experience. Non-Hispanic Black and Hispanic adults were noted to have significantly higher odds of medical mistrust compared to non-Hispanic White individuals in a survey completed prior to the COVID-19 pandemic [44], emphasizing that sociodemographic characteristics and lived experience related to other identities must be considered when evaluating the experiences of individuals with long COVID. It will be important for future research to include a demographically diverse and representative sample in order to provide comprehensive insight on the needs within long COVID support services.”* (page 27, lines 567-581)

4. The discussion references concepts like stigma, uncertainty, and existential change, but these are treated descriptively rather than fully theorized.

*Thank you for this suggestion. We have expanded on the theoretical origins and implications of these constructs in the Discussion to further enhance our understanding of the impacts of long COVID:*

*“It has also been noted that the social stigma surrounding long COVID correlates with poorer mental health and mental health-related quality of life [30]. Stigma, enabling discrimination and reducing social acceptance, has been associated with several negative impacts including impaired mental health, social relationships, and access to opportunities [31,32]. The Health Stigma and Discrimination Framework, developed to apply to a range of medical conditions including leprosy, cancer, and obesity, describes drivers of stigma (e.g., beliefs that individuals with mental health issues are dangerous), facilitators of stigma (e.g., perception that individuals with mental health issues are incompetent), as well as intersecting stigmas (such as race, gender identity, sexual orientation) [33]. These contributors to stigma impact manifestations among affected individuals (e.g., discrimination, negative public attitudes) as well as outcomes (e.g, delays in accessing care, employment restrictions, or health insurance coverage) [33].^”^* (page 23, lines 473-484)

*“The substantial uncertainty noted by our participants was similar to the consideration of medically unexplained symptoms or medical conditions without known organic cause, which are frequently associated with long COVID [35,36]. Individuals with medically unexplained syndromes and medical providers treating individuals with these conditions have frequently noted uncertainty regarding several aspects of these conditions, including causes and ideal treatment [37,38]. Individuals with long COVID note similar substantial uncertainty, affecting perceptions of their condition and expected future.”* (page 24, lines 503-519)

*”Identity loss and reconstruction as well as biographical disruption have been frequently noted in the context of chronic illness [27], and individuals with long COVID may struggle with understanding their own biography and place in the world after substantial disruptions to the body and daily life. These findings indicate that individuals with long COVID may not only struggle with immediate mental health symptoms but more existential, long-lasting doubts about their role and future in their new experience.”* (pages 21, lines 447-452)

5. Engagement with frameworks such as illness narratives, disability studies, or medical sociology could have added richer interpretive layers.

Thank you for this comment. We have expanded on how understanding long COVID through a disability studies and medical sociology lens may further explore the impact of long COVID beyond the physical symptoms:

“*The substantial and disabling nature of long COVID symptoms was frequently noted throughout the interviews. In addition to the individual impacts of symptoms, participants often noted a change in their autonomy and function, sometimes noting that they now consider themselves to have a disability. Disability can be a social identity, indicating potential implications beyond the immediate impact of physical and mental health symptoms experienced related to long COVID. Other studies in the context of long COVID have emphasized the expansive nature of disability that can affect individuals, with the impact of physical symptoms compounded by barriers to social inclusion, cognitive symptoms, challenges in completing activities of daily living, as well as mental and emotional health problems [39]. Others with long COVID have noted the social implications of long COVID as an invisible disability, noting that others often are not accepting and understanding [40], influencing social relationships and the lived experience. Long COVID has been noted to substantially affect an individual’s sense of self, often with notable identity loss [40], indicating that long COVID can impact much beyond the physical symptoms. Although some individuals with long COVID may note disability, it is also important to note that others may not identify as an individual with a disability.* (pages 25, lines 510-524)

This revised manuscript represents original work. It is not under concurrent consideration for publication nor does not contain information that is currently submitted or published elsewhere. The authors are in full control over identifying information. All authors have seen and approved this revised manuscript. No financial interests are reported.

Thank you for your reconsideration of this revised manuscript for publication in *PLOS Mental Health.*

Sincerely,

The authors
