# Supplementary material for: The lived experience of long COVID: A thematic analysis of an in-depth interview study
Source: PLOS Ment Health. 2026 Feb 6;3(2):e0000500. doi: 10.1371/journal.pmen.0000500 (PMC12880701; doi:10.1371/journal.pmen.0000500)
Supplement: Attachment — Submitted filename: longcovid_response2_4_reviewers.docx [file pmen.0000500.s025.docx]

Steve Zimmerman, PhD

Staff Editor

*PLOS Mental Health*

Dear Dr. Zimmerman,

We are submitting our revised manuscript “The lived experience of long COVID: A thematic analysis of an in-depth interview study” for reconsideration for publication in *PLOS Mental Health.* We have responded to each reviewer comment below. The authors thank the reviewers for the careful review and thoughtful comments.

Reviewer #1: Again, your theme as well as the qualitative approach is very interesting and provides new useful information. I would appreciate even more detailed description of potential bias caused by relative ethnic cultural and socio-economic homogeneity of the sample.

*Thank you for this suggestion. We have expanded on the potential implications caused by the relative sociodemographic homogeneity of the sample, emphasizing the importance of including more representative samples in future work:*

*“Our participants predominantly identified as female, white and not Hispanic or Latino/a/e, limiting interpretation to populations beyond this sample. Other work centered on long COVID has argued for the importance of promoting diversity and inclusion of diverse sociodemographic characteristics and experiences, with success incorporating community- and patient-engaged models [40,43]. Further work investigating the lived experience of long COVID from the experience of a diverse set of individuals with long COVID may provide additional insight into the variability of the impacts of long COVID. Sociodemographic characteristics such as race and ethnicity have been associated with stigma and health disparities [44], indicating the importance of considering how these factors influence the long COVID illness experience. Non-Hispanic Black and Hispanic adults were noted to have significantly higher odds of medical mistrust compared to non-Hispanic white individuals in a survey completed prior to the COVID-19 pandemic [45], emphasizing that sociodemographic characteristics and lived experience related to other identities must be considered when evaluating the experiences of individuals with long COVID. Non-white individuals and individuals without college degrees have been suggested to be more likely report long COVID, as well as activity limitations due to long COVID [46], emphasizing the potential limitations of applying our results to the wider long COVID population, given our heterogenous sample consisting mostly of white individuals with higher education. Individuals with Black and Hispanic identities have been suggested to have significantly greater odds of developing long COVID compared to white and non-Hispanic individuals, respectively [47]. Symptoms of long COVID have been suggested to vary significantly based on sociodemographic group, further emphasizing the potential limitations presented by our largely homogenous sample. The limited sociodemographic diversity within a substantial proportion of long COVID literature has been critiqued, with some noting that incorporating a more diverse set of characteristics will be important in developing a more complete picture of long COVID [48]. It will be important for future research to include a demographically diverse and representative sample in order to provide comprehensive insight on the needs within long COVID support services*.” (page 28, lines 569-595)

Since it describes the methodology rather than results, the introductive part of chapter Results should be a part of Materials and Methods.

*Thank you for pointing this out. We have moved the mentioned aspects of the Results section to the Materials and Methods:*

*“Total interview durations ranged from 38 minutes to 3 hours and 12 minutes. Participants were an average age of 51.6 (SD = 17.0) years, with most identifying as female (61.8%), white (97.1%), and not Hispanic or Latino/a/e (97.1%). Most participants had achieved at least a bachelor’s degree (79.4%), and nearly half of the participants (47.1%) reported earning greater than $100,000 per year.”* (page 7, lines 131-135)

*“The range of agreement for individual codes ranged from 82.4% to 100%, with an average agreement score of 92.5%. The Kappa (RK) scores for individual interviews ranged from 0.90 – 0.98.”* (page 9, lines 158-160)

Good luck with your future research!

*Thank you.*

This revised manuscript represents original work. It is not under concurrent consideration for publication nor does not contain information that is currently submitted or published elsewhere. The authors are in full control over identifying information. All authors have seen and approved this revised manuscript. No financial interests are reported.

Thank you for your reconsideration of this revised manuscript for publication in *PLOS Mental Health.*

Sincerely,

The authors
